# The Gut Microbiome in Autism: Study-Site Effects and Longitudinal Analysis of Behavior Change

**DOI:** 10.1128/mSystems.00848-20

**Published:** 2021-04-06

**Authors:** Jennifer Fouquier, Nancy Moreno Huizar, Jody Donnelly, Cody Glickman, Dae-Wook Kang, Juan Maldonado, Rachel A. Jones, Kimberly Johnson, James B. Adams, Rosa Krajmalnik-Brown, Catherine Lozupone

**Affiliations:** a Department of Medicine, University of Colorado, Anschutz Medical Campus, Aurora, Colorado, USA; b Computational Bioscience Graduate Program, University of Colorado, Anschutz Medical Campus, Aurora, Colorado, USA; c Department of Civil and Environmental Engineering, The University of Toledo, Toledo, Ohio, USA; d KE Genomics Core, Arizona State University, Tempe, Arizona, USA; e Biodesign Center for Fundamental and Applied Microbiomics, Arizona State University, Tempe, Arizona, USA; f Mary Bridge Children’s Developmental Behavioral Pediatrics, Tacoma, Washington, USA; g School for Engineering of Matter, Transport and Energy, Arizona State University, Tempe, Arizona, USA; h Biodesign Swette Center for Environmental Biotechnology, Arizona State University, Tempe, Arizona, USA; i School of Sustainable Engineering and the Built Environment, Arizona State University, Tempe, Arizona, USA; University of California San Diego

**Keywords:** autism, autism spectrum disorder, microbiome, microbiota, gut, next-generation sequencing, longitudinal study design, 16S rRNA gene, behavioral severity, behavior

## Abstract

Research relating gut microbiome composition to autism spectrum disorders (ASD) has produced inconsistent results, indicative of the disorder’s complexity and the need for more sophisticated experimental designs. We address this need by (i) comparing gut microbiome composition between individuals with ASD and neurotypical controls in Arizona and Colorado using standardized DNA extraction and sequencing methods at both locations and (ii) longitudinally evaluating the gut microbiome’s relationship to autism behavioral severity, diet, and gastrointestinal symptoms. Gut microbiome composition differed between individuals in Arizona and individuals in Colorado, and gastrointestinal symptoms were significantly higher in ASD individuals than in neurotypical individuals in Arizona but not in Colorado. Gut microbiome composition was significantly associated with ASD while controlling for study-site location but not when controlling for gastrointestinal symptoms. This suggests that non-ASD-related study site differences in gut microbiome composition and different degrees of gastrointestinal symptoms involvement with ASD between sites may contribute to inconsistent results in the literature regarding the association between gut microbiome composition and ASD. In the longitudinal analysis, we found that difference in levels of lethargy/social withdrawal measured in individuals at different time points correlated with the degree of change in gut microbiome composition and that a worsening of inappropriate speech between time points was associated with decreased gut microbiome diversity. This relationship between changes in the gut microbiome composition within individuals and ASD behavioral severity metrics indicates that longitudinal study designs may be useful for exploring microbial drivers of ASD severity when substantial variability exists in baseline microbiome compositions across individuals and geographical regions.

**IMPORTANCE** Autism spectrum disorder (ASD) is a brain developmental disorder with varying behavioral symptom severity both across individuals and within individuals over time. There have been promising but also inconsistent literature results regarding how the gut microbiota (microbiome) may be involved. We found that the gut microbiome in individuals with ASD is affected by study-site location as well as gastrointestinal symptom severity. When we sampled some individuals with ASD at several different time points, we found that some behaviors, such as lethargy/social withdrawal and inappropriate speech, changed along with changes in the gut microbiota composition. This is the first study to relate severity of behavior symptoms to gut microbiome composition within individuals over time and suggests a dynamic relationship between ASD-associated symptoms and gut microbes. Longitudinal study designs as well as collaborative efforts across multiple centers are needed to fully characterize the relationship between ASD and gut microbes.

## INTRODUCTION

Autism spectrum disorder (ASD) includes brain developmental disorders defined by poor socialization skills, deficiencies in communication (1), repetitive body movements (stereotyped behavior), and restricted interests (2). The Centers for Disease Control and Prevention surveyed the diagnosis of ASD in children and found that today, 1 in 54 children have ASD, compared to 1 in 150 children in 2000 (3) (https://www.cdc.gov/ncbddd/autism/addm.html). There is evidence of a link between the gut microbiome and ASD, and children with ASD have a higher incidence of gastrointestinal (GI) disorder comorbidity than that of neurotypical (NT) children (5–8). These GI problems can include abdominal pain, bloating, constipation, or loose stools (8). GI disorders within ASD cohorts have been linked with worse behavior as assessed by the aberrant behavior checklist (ABC) (9). Research demonstrated GI symptom and behavioral symptom improvements in ASD individuals with short-term antibiotic treatment (10) or through microbial transplant therapy (MTT) (11). Furthermore, investigation of the gut microbiome in a maternal infection mouse model of ASD supported a causal role for the gut microbiome in ASD symptoms, e.g., through the production of 4-ethylphenyl sulfate, a metabolite that induces anxiety (12). Another study demonstrated that ASD microbiota induce autistic-associated behavior in mice by performing gut microbiota transplants from ASD or NT human donors to gnotobiotic mice recipients (13). Additionally, increasing evidence to support a role of the gut microbiome in modulating brain signaling, which is commonly called the gut-brain-axis, is emerging (14, 15). Taken together, these studies support a causal role of the gut microbiome in ASD symptoms and suggest that the gut microbiome may be a useful therapeutic target.

One challenge in defining a role for the gut microbiome in ASD is the complexity and heterogeneity of the disorder. ASD individuals have various degrees of GI involvement, and many also have highly self-restricted diets (16), resulting in challenges when attempting to make associations with the gut microbiome. These challenges may be evident in the conflicting results published about the gut microbiome and its relationship to ASD (17). For instance, although most studies have reported differences in gut microbiome composition to occur with ASD (11, 18), some have not (19). Of those that have reported differences, there is a lack of consistency in which taxa are reported to differ (17). Such inconsistencies may be related to differences in gut microbiomes driven by other factors in the underlying etiology, in different study-site locations, or in the subtypes within the complex spectrum. A further analytic challenge is high interpersonal variation of the gut microbiome, which can lead to a scenario in which different organisms may drive different phenotypes in different individuals, despite an underlying similarity in mechanism (20).

Little is known about the stability or volatility of ASD-associated gut microbiomes over time. It is known that the severity of behavioral symptoms within autistic children can be quite variable and affected by factors such as fever, which can result in fewer aberrant behaviors (21), diet, which can have various relationships to aberrant behavior (22), or GI distress, which is associated with more aberrant behavior (5). Whether behavioral differences are associated with gut microbiome dissimilarities within ASD individuals over time is not understood and prompted us to look at the gut microbiome over multiple time points within an ASD cohort.

To assess the link between ASD and gut microbiome composition and its relationship to ASD-associated behavioral severity, diet, and GI distress, we performed targeted 16S rRNA gene sequencing of the V4 region on DNA extracted from fecal samples of children with ASD and from those of NT controls. Our study design addresses two analytic issues that have challenged a consistent picture to emerge on the degree and nature of gut microbiome differences that occur with ASD. First, we investigated if different geographic locations may underlie inconsistent gut microbiome associations with ASD by analyzing a combined cohort of ASD and NT control individuals recruited in Arizona (AZ) and Colorado (CO). Second, we collected 2 to 4 samples longitudinally (span of 3 to 13 months apart) from 16 individuals in CO (7 with ASD and 9 non-ASD controls) with information on ASD-associated behavioral severity, diet, and GI symptoms at each collection, so that relationships between these factors within individuals and over time could be assessed. Taken together, our results support that gut microbiome differences occur between ASD and NT but that these differences are more subtle compared to differences in study-site location and may be affected by GI symptoms. Longitudinal analysis within ASD individuals supports a relationship between differences in the gut microbiome composition and ASD-associated behaviors over time and within individuals.

## RESULTS

We first sought to identify differences in the gut microbiome that may occur between individuals with ASD and NT controls using data from cohorts recruited in AZ and CO (summarized in Table 1). The AZ cohort consists of a total of 74 individuals described in previous studies (23, 24): 36 children with ASD and 38 age-matched NT controls who did not have first-degree relatives with autism. The CO cohort consisted of 29 individuals, 13 children with ASD and 16 age-matched NT controls, of which 5 were siblings of individuals with ASD and 11 were unrelated. Within the CO cohort, 7 ASD individuals and 9 NT controls were studied over time, with each individual providing 2 to 4 samples spaced an average of 6 months (standard deviation [SD] = 3 months) apart (span of 3 to 13 months; Table 1), together with information on their behavioral severity, recent diet, and GI symptoms with each collected sample. For our cross-sectional analysis of both cohorts together, only one time point sample from each individual in the CO cohort was selected as described in Materials and Methods.

**TABLE 1:** Characteristics of cohort subgroups

Cohort	Diagnosis	No. of individuals	Female (%)	Male (%)	Age mean	Age SD	Mean GI severity (scale 0–4)	No. of individuals with longitudinal data (2–4 samples)
Colorado	ASD	13	15.38	84.62	6.85	2.51	0.67	7
	Control: sibling to someone with ASD	5	40	60	4.6	1.52	0.25	3
	Control: unrelated	11	54.55	45.45	6.36	1.63	0	6
Arizona	ASD	36	8.33	91.67	8.47	3.86	1.97	0
	Control: unrelated	38	23.68	76.32	8.61	3.92	0.68	0

Although the AZ and CO studies were conducted by different research groups (led by Rosa Krajmalnik-Brown and James Adams in AZ and Catherine Lozupone in CO), efforts were made to standardize how the fecal samples were collected, processed, and sequenced. Specifically, fecal samples in both AZ and CO were collected at home by study participants and shipped frozen. The same DNA extraction protocol was used at both sites (MoBio PowerSoil DNA isolation kit, Qiagen, Venlo, Limburg, NL), and DNA extracts were multiplexed and sequenced together in AZ. Furthermore, a subset of samples had DNA extractions performed in both AZ and CO to confirm that differences between laboratories did not drive differences observed between locations.

### Cross-sectional analysis.

The cross-sectional analysis was composed of 103 individuals (74 from AZ and 29 from CO), 49 with ASD and 54 NT. Sequencing of the V4 region of the 16S rRNA gene region was performed on extracted DNA on the Illumina MiSeq platform at the Microbiome Facility at the Biodesign Institute (https://biodesign.asu.edu/microbiome-facility), Arizona State University. AZ and CO both collected information on GI symptoms but used different questionnaires as detailed in Materials and Methods. Responses to these questionnaires were standardized for comparison across locations by assigning a sub score to the frequency responses from the CO GI symptom questionnaire using the same scale as that used by the AZ GI symptom questionnaire for constipation, diarrhea, and abdominal pain.

The CO cohort (age range 2 to 11 years) was significantly younger than the AZ cohort (age range 3 to 17 years; mean 6.28 years for CO versus 8.54 years for AZ, *P = *0.014). The ASD cohort had significantly more GI issues than the unrelated controls in AZ (Dunn [1964] Kruskal-Wallis test, *P < *0.001) but not in CO. The ASD cohort in AZ also had more GI issues than the ASD cohort in CO (Dunn [1964] Kruskal-Wallis test, *P = *0.02) (Table 2). This was the case even though the presence and/or severity of GI symptoms was not considered during subject recruitment at either site. The AZ study evaluated their individuals’ symptoms on a weekly basis, while the CO study evaluated their individuals’ symptoms on a monthly basis. Even after controlling for differences in the GI survey administration using normalization efforts that are explained in Materials and Methods, AZ individuals had more severe GI symptoms compared to those of CO individuals.

**TABLE 2:** Comparison of gastrointestinal symptom severity by location and ASD status

Sample group	Z	*P* value	Adjusted *P* value
AZ ASD individuals^*a*^
CO ASD individuals	2.75	<0.001	0.02
CO sibling controls
AZ ASD individuals	2.56	0.01	0.03
CO ASD individuals	0.75	0.45	0.65
AZ unrelated controls
AZ ASD individuals	4.33	<0.001	<0.001^*b*^
CO ASD individuals	0.28	0.78	0.78
CO sibling controls	−0.65	0.52	0.64
CO unrelated controls
AZ ASD individuals	4.86	<0.001	<0.001^*b*^
CO ASD individuals	1.82	0.07	0.12
CO sibling controls	0.56	0.58	0.64
AZ unrelated controls	1.95	0.05	0.10

a All subheadings in the first column are compared to each indented subgroup.

b Test resulted in the ASD group having significantly more GI issues than unrelated controls in AZ (*P *< 0.001) and ASD group in AZ having significantly more GI symptoms than the unrelated controls in CO (*P* < 0.001) using Dunn (1964) Kruskal-Wallis test.

### Gut microbiota composition is influenced by study site and gastrointestinal symptoms.

**FIG 1:**
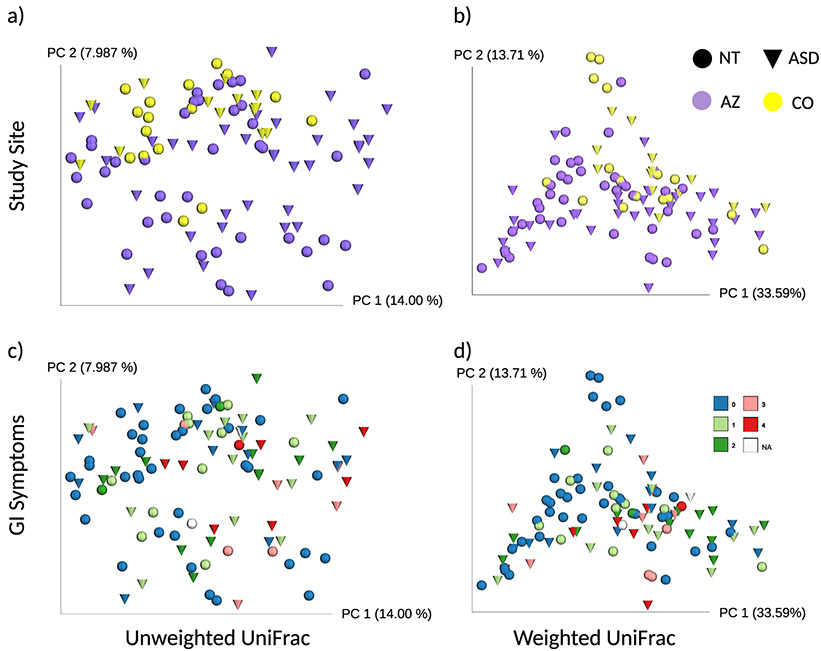
PCoA plots of unweighted and weighted UniFrac distances visualized by study site, ASD status, and GI symptoms. (a) Unweighted UniFrac distances and (b) weighted UniFrac distances show samples clustering by location and not ASD status. CO is yellow and AZ is purple. AZ had greater beta diversity than did CO due to the greater spread of samples across the plot. There was a significant effect of study site on unweighted UniFrac distance variability but not for weighted UniFrac distances (PERMDISP, unweighted UniFrac: F-value = 11.7, *P* = 0.002; weighted UniFrac: F-value = 2.6, *P* = 0.142). Study-site location clustering had the largest effect when using Adonis (a) unweighted UniFrac: *P = *0.001, *R^2^* = 0.029; (b) weighted UniFrac: *P = *0.001, *R^2^* = 0.054, followed by GI symptoms (c) unweighted UniFrac: *P = *0.025, *R^2^* = 0.016; (d) weighted UniFrac: *P = *0.006, *R^2^* = 0.033 (see Results for detailed explanation). GI symptoms range from 0 to 4, where 4 indicates the most severe GI symptoms and is colored red. Two individuals did not complete the GI survey (NA values in white). ASD, autism spectrum disorder; NT, neurotypical; AZ, Arizona; CO, Colorado; GI, gastrointestinal.

There was a significant effect of site location (AZ versus CO) and GI symptoms on gut microbiome composition. Site location had the greatest effect on gut microbiome beta diversity (Fig. 1; Adonis, unweighted UniFrac: *P = *0.001, *R^2^* = 0.029; weighted UniFrac: *P = *0.001, *R^2^* = 0.054), followed by GI symptoms (Fig. 1; Adonis, unweighted UniFrac: *P = *0.025, *R^2^* = 0.016; weighted UniFrac: *P = *0.006, *R^2^* = 0.033). ASD was not significant after controlling for both site location and GI symptoms, but it was significant while controlling only for site location (Adonis, unweighted UniFrac: *P* = 0.009, *R^2^* = 0.019; weighted UniFrac: *P = *0.017, *R^2^* = 0.025). An effect of the study site but not of ASD was clearly evident with principal coordinates analysis (PCoA) with both unweighted and weighted UniFrac (Fig. 1A and B). Individuals from AZ showed greater beta diversity than that of individuals from CO, as seen by a greater spread of points across PC2 of samples from AZ in both unweighted and weighted UniFrac PCoA analysis (Fig. 1A and B). Indeed, study site had a significant effect on unweighted UniFrac distance variability but not for weighted UniFrac distances (PERMDISP, unweighted UniFrac: F-value = 11.7, *P* = 0.002; weighted UniFrac: F-value = 2.6, *P* = 0.142).

The visible clustering by study site was surprising, since methods for fecal sample collection were highly similar between sites, the same DNA extraction method was used (though applied in different labs), and sequencing of the samples from both sites was done together. To further explore whether methodological differences between sites could influence the observed microbial diversity, 9 samples from CO were sent to AZ for reextraction. Adonis indicated that there was no significant effect of DNA extraction location on microbial diversity (Adonis, unweighted UniFrac: *P* = 0.226, weighted UniFrac: *P* = 0.160, Fig. S1). Additionally, the average beta diversity distances between the same stool sample sequenced twice, with DNA extracted in CO and AZ (mean unweighted UniFrac distance: 0.18; mean weighted UniFrac distance: 0.12), were significantly lower than beta diversity distances between unique stool samples collected from the same individual at different time points (mean unweighted UniFrac distance: 0.41; mean weighted UniFrac distance: 0.23) (Mann-Whitney test, unweighted UniFrac: *P* < 0.0001 and weighted UniFrac: *P* = 0.010). Although the extractions conducted at different sites did have some qualitative differences in the gut microbiome, these could not explain the differences observed between locations.

10.1128/mSystems.00848-20.1 FIG S1 Unweighted and weighted beta diversity PCoA plots for nine fecal samples from individuals from Colorado extracted in both Arizona and Colorado. (A) Unweighted UniFrac PCoA plot and (B) weighted UniFrac PCoA plots. Adonis indicated that there was no significant effect of DNA extraction location on microbial diversity (Adonis: unweighted UniFrac, *P* = 0.226; weighted UniFrac, *P* = 0.160). Spheres are samples extracted in Arizona and squares are the same sample extracted in Colorado. ASD, autism spectrum disorder; PCoA, principal coordinates analysis. Download FIG S1, PDF file, 0.7 MB. Copyright © 2021 Fouquier et al. 2021 Fouquier et al. https://creativecommons.org/licenses/by/4.0/ This content is distributed under the terms of the Creative Commons Attribution 4.0 International license.

Although GI symptoms only explained a small (but statistically significant) proportion of beta diversity with Adonis, it is notable that the observed operational taxonomic units (OTUs) alpha diversity metric (which is the number of different OTUs observed in a sample at a standardized sampling depth) strongly correlated with variability across PC1 with unweighted UniFrac (*R*^2^ = −0.87; *P* = <0.001, Pearson’s product-moment correlation; Fig. S2A and B), but alpha diversity did not have a significant association with ASD status in this study. AZ consisted of individuals with both large and small amounts of observed OTUs, but CO did not have samples that were as low in observed OTUs as those in AZ.

10.1128/mSystems.00848-20.2 FIG S2 PCoA plot of unweighted UniFrac distance metric and correlation between PC1 and observed OTUs. (A) Samples range from high numbers of observed OTUs to low numbers of observed OTUs along principal coordinate axis one. (B) Correlation between PC1 coordinate values from unweighted UniFrac distance matrix and observed OTUs. Yellow markers are for Colorado and purple markers are for Arizona. Triangles represent ASD individuals and circles represent neurotypical controls. ASD, autism spectrum disorder; PC1, principal coordinate one. Download FIG S2, PDF file, 0.2 MB. Copyright © 2021 Fouquier et al. 2021 Fouquier et al. https://creativecommons.org/licenses/by/4.0/ This content is distributed under the terms of the Creative Commons Attribution 4.0 International license.

Since the ASD cohort in AZ had more GI issues than the ASD cohort in CO, we also wondered whether the location effect could be driven by higher GI issues in the AZ-ASD cohort. However, a study-site effect was still evident when only considering samples from unrelated non-ASD controls who did not have significantly different levels of GI symptoms (unweighted UniFrac: *P = *0.002, *R^2^* = 0.048; weighted UniFrac: *P = *0.002, *R^2^* = 0.112).

### Random forest classification of autism spectrum disorder status.

**FIG 2:**
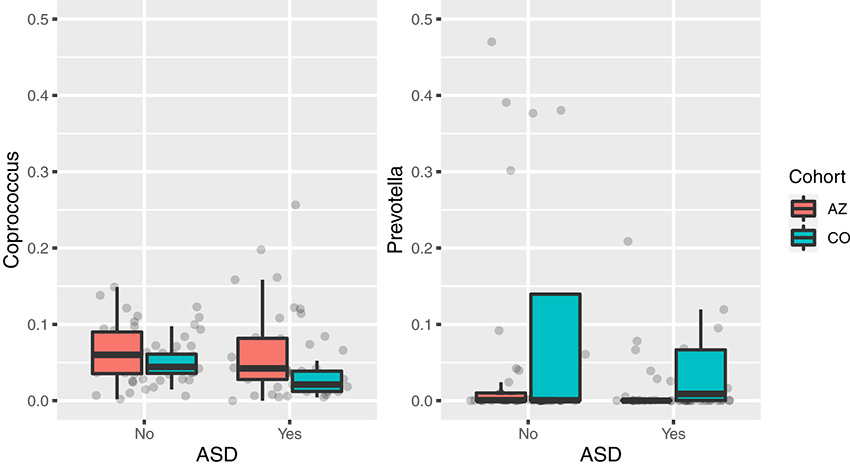
Relative abundance of *Coprococcus* and *Prevotella* for each study-site location in individuals with and without ASD. Box and whisker plots are colored by study site and grouped by ASD status with *Coprococcus* on the left and *Prevotella* on the right. Individuals in CO had lower levels of *Coprococcus* (Kruskal-Wallis, χ*^2^* = 4.17, *P = *0.041) and higher levels of *Prevotella* (Kruskal-Wallis, χ*^2^* = 6.18, *P = *0.013) compared to those of individuals in AZ. ASD status was not significantly related to relative abundance of either genus. AZ, Arizona; CO, Colorado; ASD, autism spectrum disorder.

After analyzing whether extraction site or higher GI issues in AZ were possible explanations for the study-site differences, we sought to understand whether bacterial genera (as assigned with the Greengenes database 13.8 [25, 26]) or amplicon sequence variants (ASVs; defined using DADA2 [27]) could differentiate individuals with ASD from NT controls using Random Forest. A classifier was built using samples from both AZ and CO, which yielded an area under the curve (AUC) of 0.83 for genera and 0.86 for ASVs, better than random chance (AUC = 0.5). The highest Random Forest importance values greater than or equal to 0.01 resulted in 13 ASVs for one classifier and 41 genera for the other classifier (Table S3). These important features were further evaluated with a linear model. This model was used to assess the impact of ASD, GI scores, and changes between AZ and CO on each microbial taxon, although after false-discovery rate (FDR) correction, no significant relationships were identified for ASD status, GI scores, or study-site location (Table 3). A previous study had identified decreases in *Coprococcus* and *Prevotella* genera with ASD status in AZ (24), so we specifically interrogated for changes in these taxa because they were also found among the most important features (Table S3). Individuals in CO had a lower relative abundance of *Coprococcus* (Kruskal-Wallis: χ*^2^* = 4.17, *P = *0.041) and higher relative abundance levels of *Prevotella* (Kruskal-Wallis, χ*^2^* = 6.18, *P = *0.013) than those of AZ samples overall, while the relative abundance of these genera did not differ by ASD status (Kruskal-Wallis: *Coprococcus*, *P = *0.093; *Prevotella*, *P* = 0.058) (Fig. 2 and Table 3). These differences between AZ and CO motivated building a second classifier with only AZ samples and evaluating if this classifier could accurately predict ASD status in CO samples. The model predicted samples from AZ with an AUC of 0.66 and samples from CO with an AUC of 0.60, indicating that while location-specific classifiers predict ASD status less accurately, combining samples from both locations serves as a greater predictor.

**TABLE 3:** Random Forest importance values and linear model independent variable importance on individual genus level microbial taxa^*a*^

Family, genus	Random Forest importance	Variable	*P* value	*t* value
*Prevotellaceae*, *Prevotella*	0.014476193	ASD	0.188	−1.32
		GI score	0.673	−0.42
		Change between AZ and CO	0.002	3.19
*Lachnospiraceae*, *Coprococcus*	0.018451293	ASD	0.449	−0.76
		GI score	0.579	0.56
		Change between AZ and CO	0.126	−1.54

a A negative *t* value indicates lower levels of a microbe in individuals with ASD.

10.1128/mSystems.00848-20.8 TABLE S3 Important features from ASV and Genus Random Forest classifiers created using combined Arizona and Colorado samples. Most important features are at the top and listed in descending order using a cutoff of 0.01. Download Table S3, DOCX file, 0.02 MB. Copyright © 2021 Fouquier et al. 2021 Fouquier et al. https://creativecommons.org/licenses/by/4.0/ This content is distributed under the terms of the Creative Commons Attribution 4.0 International license.

### Longitudinal analysis.

As indicated by our cross-sectional analyses, an individual’s gut microbiome can be influenced by factors such as study-site location or GI symptoms. These various gut microbiome compositions can affect cross-sectional analyses, hiding the effects of other factors such as ASD. By using each individual as their own control with their own baseline gut microbiome, we used a longitudinal study design to understand how changes in the gut microbiome may relate to changes in the severity of ASD-associated behaviors in children with ASD. We collected between 2 and 4 stool samples from 7 ASD individuals and 9 NT controls from CO (Table 1). AZ individuals did not participate in the longitudinal study. Stool samples were collected an average of 6 months (SD = 3 months) apart. At the time of each fecal sample collection, study participants also filled out questionnaires to assess their diet within the prior week using the Block Kids food frequency questionnaire (FFQ), GI symptoms over the last 3 months or time since the last sample collection, and behavior over the last 4 weeks using the ABC for those with ASD (for survey details see Materials and Methods).

### The gut microbiome shows a high degree of interpersonal variation and variability over time.

**FIG 3:**
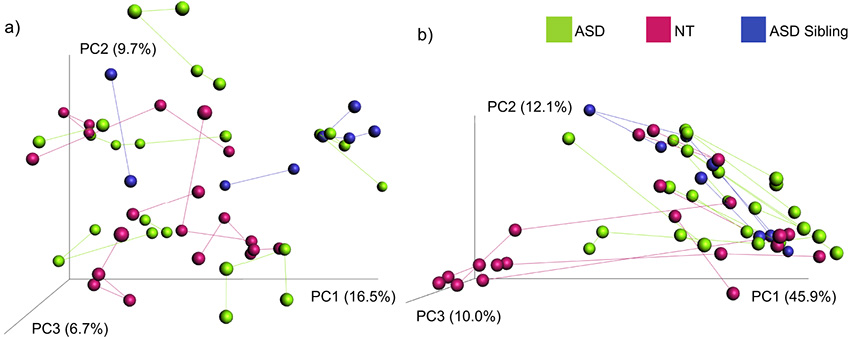
Beta diversity PCoA plots for longitudinal gut microbiome samples from ASD individuals, NT control individuals, and ASD siblings. Beta diversity analysis PCoA plots with (a) unweighted UniFrac and (b) weighted UniFrac where individual gut microbiome samples of ASD, NT control, and sibling to someone with ASD are connected by vectors. PCoA, principal coordinates analysis; ASD, autism spectrum disorder; NT, neurotypical.

The gut microbiome showed a high degree of interpersonal variation, with beta diversity between samples taken from the same individual at different time points being significantly smaller than beta diversity between samples taken from different individuals and with “individual” accounting for 5.61% variation for unweighted UniFrac and 8.95% for weighted UniFrac (Fig. 3, unweighted UniFrac, Adonis, *P < *0.001, *R^2^* = 5.61%; weighted UniFrac, Adonis, *P = *0.01, *R^2^* = 8.95%). Beta diversity on different days between ASD individuals, NT individuals, and ASD siblings was not significantly different using the Kruskal-Wallis test, whether siblings with ASD were included in the neurotypical control group (unweighted UniFrac, *P = *0.71; weighted UniFrac, *P = *0.12) or analyzed as their own group (Fig. S3; unweighted UniFrac, *P = *0.57; weighted UniFrac, *P = *0.23). Qualitative observations indicated that there was more gut microbiome stability between sampling days one and two within ASD individuals than within controls (Fig. S3), indicating that this might be of value to explore in a larger cohort with more statistical power.

10.1128/mSystems.00848-20.3 FIG S3 Pairwise distance boxplots comparing beta diversity changes within individuals. Beta diversity changes between sampling days one and two were assessed for 16 individuals using (A) unweighted UniFrac distances and (B) weighted UniFrac distances for ASD individuals, NT individuals, and ASD siblings. ASD, autism spectrum disorder; NT, neurotypical. Download FIG S3, PDF file, 1.5 MB. Copyright © 2021 Fouquier et al. 2021 Fouquier et al. https://creativecommons.org/licenses/by/4.0/ This content is distributed under the terms of the Creative Commons Attribution 4.0 International license.

We also plotted differences in gut microbiome beta diversity within individuals against time and between samples (Fig. S4). This plot did not indicate systematic directional changes in the gut microbiome with time but rather variation within a compositional space (linear mixed effect [LME] model: unweighted UniFrac, *P = *0.43; weighted UniFrac, *P = *0.35).

10.1128/mSystems.00848-20.4 FIG S4 Gut microbiome beta diversity over change in time for ASD and NT individuals. Scatterplots showing changes in (a) unweighted UniFrac distance and (b) weighted UniFrac distance over change in time, in months, from sample collection start. Cool colors represent samples from different ASD individuals, and warm colors represent samples from different neurotypical individuals. ASD, autism spectrum disorder; NT, neurotypical. Download FIG S4, PDF file, 0.03 MB. Copyright © 2021 Fouquier et al. 2021 Fouquier et al. https://creativecommons.org/licenses/by/4.0/ This content is distributed under the terms of the Creative Commons Attribution 4.0 International license.

### Linear mixed effect models for longitudinal analysis.

Next, we tested whether ASD-associated behavioral severity, diet, or GI symptoms could explain differences in the gut microbiome measured at different time points. Overall, we reasoned that if ASD-associated behavioral severity, diet, or GI symptoms either influenced or were influenced by gut microbiome composition, the degree of change in these measurements within an individual across two time points would be correlated with the degree of change in the gut microbiome across these same two time points. We assessed change in gut microbiome composition with qualitative (unweighted UniFrac [28]) and quantitative (weighted UniFrac) beta diversity metrics and several alpha diversity metrics. We evaluated changes in alpha diversity as change across time points in the following four alpha diversity measures: Pielou’s measure of species evenness (29), which is how even each species’ count is within a sample, Faith’s PD, a measure of the phylogenetic diversity of species present in a sample (30, 31), observed OTUs, which is the number of different OTUs observed in a sample at a standardized sampling depth and is a measure of richness, and Shannon Diversity (32), a diversity measure that accounts for both the OTU richness and the OTU evenness of a sample (33). We used Euclidean distance matrices (EDMs) for combined ASD-associated behavioral severity scores for ASD individuals, dietary measures for both ASD and NT individuals, and GI symptoms for both ASD and NT individuals. We compared these EDMs to qualitative and quantitative gut microbiome beta diversity and change in alpha diversity. The change in (delta) score for individual ASD-associated behavioral severity metrics was used to compare these metrics to beta diversity metrics. The raw scores for ASD-associated behavioral severity were used to compare behavior to the alpha diversity metrics.

Since there was no significant relationship between time between measurements and microbiome diversity (Fig. S4), we used all pairwise comparisons within each individual in LME models even though the samples may have been collected for variable amounts of time apart. For all analyses that included an EDM, a vector containing only pairwise comparisons between samples from each individual was used. This allowed for gut microbiome relationships to be identified for each individual, independent of how that individual’s sample’s gut microbiome compared to another individual’s sample’s gut microbiome. Because no distance matrices (sample comparisons) were involved when assessing relationships between ASD-associated behavioral severity metrics and alpha diversity metrics, the original scores were used for these linear models.

### Autism-associated behavioral severity is associated with differences in the microbiome at different time points.

First, we evaluated whether there was a positive relationship between changes in reported ASD-associated behavioral severity and changes in gut microbial beta diversity, over time and between only the ASD individuals’ samples. The ABC provides measures of 5 different aspects of behavior: hyperactivity, inappropriate speech, irritability, lethargy/social withdrawal, and stereotypy. These five individual ASD-associated behavior metrics, as well as an EDM for overall ABC scores, were related to unweighted UniFrac distances and weighted UniFrac distances using pairwise distances or changes between samples within ASD individuals. The five individual ASD-associated behavior metrics were compared to raw alpha diversity values. ABC EDM was additionally compared to changes in the four alpha diversity metrics.

**FIG 4:**
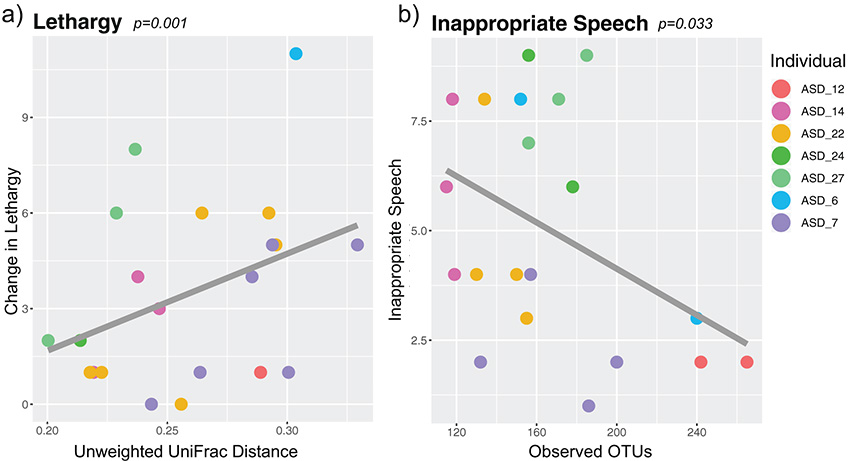
Relationship between autism-associated behavioral severity and gut microbiome beta diversity and alpha diversity in seven ASD individuals over time, using linear mixed effects models. Relationship between beta diversity, using unweighted UniFrac distances, and (a) change in lethargy/social withdrawal (LME estimate: 65.72; likelihood ratio test: *P = *0.001, *q *= 0.006, χ*^2^* = 10.43) or (b) relationship between alpha diversity, using observed OTUs, and inappropriate speech (LME estimate: −0.036; likelihood ratio test: *P = *0.033, *q *= 0.163, χ*^2^* = 4.57). ASD, autism spectrum disorder; OTU, operational taxonomic units; LME, linear mixed effects. Statistics summary found in Table S2.

There was a significant, positive correlation between change in ASD lethargy/social withdrawal and unweighted UniFrac distance when comparing full to reduced models using likelihood ratio tests (LME estimate: 65.72; likelihood ratio test: *P = *0.001, *q *= 0.006, χ*^2^* = 10.43) (Fig. 4A and Table S2).

10.1128/mSystems.00848-20.7 TABLE S2 Summary of linear mixed effect models (LMEs) relating autism behavioral severity metrics, GI distress, and diet to unweighted UniFrac and weighted UniFrac beta diversity microbiome metrics and Shannon diversity, observed OTUs, Evenness, and Faith’s PD alpha diversity microbiome metrics. Download Table S2, DOCX file, 0.02 MB. Copyright © 2021 Fouquier et al. 2021 Fouquier et al. https://creativecommons.org/licenses/by/4.0/ This content is distributed under the terms of the Creative Commons Attribution 4.0 International license.

For alpha diversity metrics, observed OTUs had a significant negative relationship to ASD inappropriate speech when comparing full to reduced models using likelihood ratio tests (LME estimate: −0.036; likelihood ratio test: *P = *0.033, *q *= 0.163, χ*^2^* = 4.57) (Fig. 4B and Table S2) but not to the other ASD-associated behavioral severity metrics. Pielou’s evenness, Faith’s PD, and Shannon diversity did not have any significant relationships to ASD-associated behavioral severity metrics. No individual taxa varied significantly with behavior metrics after multiple comparison corrections.

### Diet and its relationship to the gut microbiome.

Dietary EDMs as assessed by the FFQ for dietary carbohydrates, daily totals, percentages, and fiber (see Materials and Methods) were used with LME models to find relationships to changes in unweighted UniFrac distances and weighted UniFrac distances for beta diversity metrics as well as changes in Pielou’s evenness, Faith’s PD, observed OTUs, and Shannon diversity for alpha diversity metrics, for both ASD individuals and NT individuals. For NT individuals, but not for ASD individuals, the dietary percentage EDM was significantly and positively related to change in Shannon diversity (Fig. S5 and Table S2; LME estimate 15.76; likelihood ratio test: *P = *0.004, *q *= 0.016, χ*^2^* = 8.31). All other dietary relationships were insignificant with respect to their relationship to the gut microbiome.

10.1128/mSystems.00848-20.5 FIG S5 Relationship between change in dietary percentages and Shannon diversity in neurotypical individuals. A dietary percentage Euclidean distance matrix was created from percentage of fat, protein, carbohydrates, and sweets for the past week (see Materials and Methods) and compared to change in Shannon diversity using linear mixed effects models. Linear mixed effects estimate 15.76; likelihood ratio test: *P = *0.004, *q *= 0.016, χ*^2^* = 8.31. Download FIG S5, PDF file, 0.01 MB. Copyright © 2021 Fouquier et al. 2021 Fouquier et al. https://creativecommons.org/licenses/by/4.0/ This content is distributed under the terms of the Creative Commons Attribution 4.0 International license.

### Gastrointestinal symptoms and their relationship to the gut microbiome.

Because ASD individuals have a high incidence of GI symptom comorbidity, we looked at changes in GI symptoms using an EDM created from reported abdominal pain, bloating, loose stool, and constipation and then compared these to changes in gut microbial diversity. We found no significant relationships using LME models for ASD or NT individuals (Table S2).

### Autism spectrum disorder associated behavioral severity and its relationship to gastrointestinal symptoms and diet.

To explore whether ASD-associated behavioral severity may be affected by fluctuations in different dietary measures or level of gastrointestinal severity, we compared ASD-associated behavioral severity to the EDMs for GI symptoms, dietary percentages, dietary daily totals, dietary fiber, and percent carbohydrates using LMEs. We found no significant results between ASD-associated behavior and GI symptoms or diet (Table S2).

## DISCUSSION

We discovered that in the cohorts used for this analysis, study-site location (whether subjects were recruited in AZ or CO) had a stronger relationship to the gut microbiome than did ASD status. Geographical differences in microbiomes have been a subject of recent research interest. The American Gut Project found a statistically significant, but weak, effect of geography on the microbiome within the US, with distance-decay relationships reaching significance at neighborhood sizes of 100 km to 1,000 km and with significantly higher alpha diversity in the US than that in Europe (34). In another study conducted on links between the microbiome and metabolic disease in 14 districts within 1 province in China, location strongly associated with microbiome composition, and models that used microbiome information to predict metabolic disease that developed in 1 location failed when used in other locations (35). Large multilocation experimental designs may be essential for robust determination of microbiome associates with complex disease or disorder. An example of such research efforts is the TEDDY study, which uses 6 clinical centers (3 in the US and 3 in Europe) to determine early like microbiome type I diabetes determinants (36). It is important to note that there are many underlying factors, such as diet, climate, or lifestyle, that might elucidate the geographical differences we identified in the gut microbiome.

Our Random Forest results support that different gut microbiome features may differentiate individuals with ASD from NT in AZ versus CO. A classifier built with AZ only can predict ASD status 16% more accurately than random chance when used on individuals in AZ who were not used to build the classifier, while prediction was only 10% better than random chance when used on CO samples. Consistent with this, differences in key taxa reported previously to differ in ASD in AZ, such as *Prevotella* (24), were not reproduced in CO, although a decrease in *Coprococcus* with ASD did appear to be consistent. CO samples have higher *Prevotella* counts than do AZ samples. This may be due to many factors, including regional dietary differences, because *Prevotella* is known to be linked to higher fiber diets (37) or frequency of exercise, as *Prevotella* has also been shown to be increased in competitive cyclists (38). Unfortunately, our AZ cohort did not include dietary survey information and neither cohort assessed physical activity, so we could not test these hypotheses directly. Overall, our Random Forest results show that gut microbiome data from one study site do not classify ASD status sufficiently beyond random chance, but including data from both sites created excellent classifiers. However, its current generalizability to other cohorts is not understood. Further multicenter research or meta-analysis would be necessary to establish whether microbiome classifiers for ASD status prediction can be accurate across diverse cohorts.

One notable difference in ASD cohorts is an increase in GI symptoms with ASD in AZ and not in CO. This occurred even though neither study considered GI symptoms in recruitment, reflecting that the population of ASD individuals sampled in AZ may have more GI involvement than those in CO, perhaps reflecting underlying heterogeneity in ASD symptoms and comorbidity in different clinics. This again supports multicenter experimental designs that also carefully characterize symptoms and comorbidity in patient populations.

An additional difference between study design was that CO and not AZ included siblings of the ASD individual in the neurotypical control group. In general, studies of ASD have differed in whether they have (19, 39, 40) or have not (24, 41) used neurotypical siblings of ASD individuals as controls. An argument for using siblings as controls is that there will be a greater similarity in other factors that may influence the microbiome independently of ASD, such as diet, other exposures, and genetics. However, a 2019 meta-analysis found that individuals who have siblings with ASD are more likely to have impaired functioning than individuals who have siblings without ASD (42), providing support for excluding siblings of ASD individuals from being controls as other studies have done (24, 41). Interestingly, one study that included both sibling and nonsibling neurotypical controls found the sibling controls to have a microbiome more similar to that of children with ASD than to that of control subjects without a neurotypical sibling (40), again providing insight into how experimental design can influence differences in the degree and nature of ASD that have been reported across studies.

To address some of the challenges created by each individual having their own unique gut microbiome composition, we used a longitudinal study design. Longitudinal study designs are common in microbiome research, and these study designs usually include assessment of microbiome differences that occur in response to an intervention or experimental manipulation, such as response to drugs or diet (43, 44). However, for many different diseases with a known/suspected link with the microbiome, disease symptoms vary over time, necessitating the approach we applied here that related changes in ASD-associated behaviors within individuals, over time, to changes in the microbiome. This approach addresses the challenge that because of high interpersonal variation in the gut microbiome across individuals, different microbes may be important for ASD symptoms in different individuals, in particular if those individuals express different behavioral phenotypes across the broad spectrum of autism, different comorbidities such as the degree of GI involvement, or different dietary triggers that may also influence the microbiome.

Longitudinal sampling of the same individual showed within-subject variation over time to be lower than between-subject variation, which is consistent with the results of many other research studies (45–47). We did not find a positive relationship with the amount of time between sample collection and the amount of difference in gut microbiome composition (beta diversity) over our sampling period of 3 to 13 months, suggesting stochastic variation around a common core rather than directed drift of the community (deterministic process) over time across this time scale, which is consistent with other studies (48). We had suspected that ASD and NT controls may differ in beta diversity over time, for instance, if restricted eating caused greater stability or GI disturbances caused a greater volatility. Indeed, greater volatility of the gut microbiome over time has been linked with disease in other systems, such as increased risk of infection in cancer patients and inflammatory bowel disease (49). However, although the mean beta diversity over time did not differ significantly between ASD and NT control individuals, we note that our study only looked at a small number of individuals and that it is possible that we did not have the statistical power to support observed trends.

Although our study cohort was small, we found several relationships between ASD-associated behavioral severity and the gut microbiome among the five aspects of behavior that we measured with the ABC: hyperactivity, inappropriate speech, irritability, lethargy/social withdrawal, and stereotypy. We identified that worsening lethargy/social withdrawal behavior within ASD individuals had a positive relationship to changes in gut microbiome beta diversity. More specifically, changes in lethargy/social withdrawal were greater between time points that had a larger shift in gut microbiome composition. A study by Sappok et al. showed that ASD was the major, and only, factor out of 10 mental and neurological disorders associated with stereotypic and lethargy/social withdrawal behaviors reported on the ABC (50), with social withdrawal and stereotypic behavior being two of the three core symptoms of autism. Additionally, a meta-analysis identified that supplementation of omega 3 fatty acids may improve hyperactivity, lethargy/social withdrawal, and stereotypy in ASD individuals (51). In a fecal microbiota transplant trial, all five behavior measurements on the ABC, including lethargy/social withdrawal and inappropriate speech, were improved together with changes in gut microbiota (11). Together, these three previous studies, combined with ours, possibly demonstrate an important relationship between lethargy/social withdrawal behavior, ASD, and the gut microbiome.

In addition to lethargy/social withdrawal, inappropriate speech in the same individual at different time points worsened when the number of gut bacteria (observed OTUs) decreased across those same time points. This trend suggests that an increase in the number of bacteria present in one’s gut microbiome may be related to improved speech in autistic individuals. This is interesting because the richness of the human gut microbiome is generally linked to health, including in attention deficit hyperactive disorder (ADHD), another disorder linked with inappropriate speech (52–55). Together, these findings suggest that shifts in the gut microbiome are related to changes in ASD-associated behavioral severity.

Interestingly, we did not find any significant relationships between changes in behaviors and changes in overall GI symptoms or diet percentages/daily totals within ASD individuals. This lack of association between behavior and diet or GI symptoms is corroborated by a 2013 cross-sectional study of 20 ASD individuals (24) and is in contrast with a previous paper in which GI symptoms were linked with worse behavior as assessed by the ABC (9). However, the individuals in CO with ASD did not have worse GI symptoms compared to those of NT controls, suggesting that these associations may not have been present because of a lack of GI disturbance. It is interesting, however, that we found a relationship between the microbiome and behaviors in the absence of GI disturbance. Future longitudinal studies that include individuals with ASD both with and without concurrent GI issues are warranted.

For neurotypical individuals, we did identify a positive relationship between change in dietary percentages and alpha diversity between sampling days, but we did not observe this in ASD individuals. Diet is an accessible way to modify the microbiome, but our findings suggest that neurotypical individuals’ microbiomes may be more amenable to dietary modification than those of autistic individuals.

Our inability to find significant relationships between individual taxa and ASD-associated behavior may be a consequence of variation in individual response to these organisms. It is also possible that low power contributed to this negative result and that a larger study may elucidate relationships between individual taxa and autism behavioral severity.

### Conclusions.

The complex nature of ASD has led to inconsistencies in published findings connecting ASD to the gut microbiome. When comparing cohorts of individuals with ASD in AZ and CO using cross-sectional analysis, we determined that study-site location had a stronger relationship to the gut microbiome than did ASD status, including in key taxa previously reported to differ in AZ, such as higher levels of *Prevotella* in CO compared to those in AZ independent of ASD status. Additionally, we identified an increase in gastrointestinal symptoms in ASD individuals in AZ but not in those in CO. Taken together, these results suggest that geographical differences in gut microbiome composition and differences in levels of gastrointestinal symptoms in autistic individuals at different study sites may contribute to inconsistent results in the literature. By performing a longitudinal analysis to understand microbiome changes within ASD individuals over time, we identified relationships between ASD-associated behavior and the gut microbiome but not between GI symptoms or diet and the gut microbiome, supporting a role for the microbiome in ASD-associated behavior severity. Multicenter studies in geographically distinct areas with more study participants, as well as longitudinal study designs that consider individual differences in the gut microbiomes of ASD individuals, are essential for a disorder of this prevalence.

## MATERIALS AND METHODS

### Recruitment and sample collection.

**Colorado.** Children diagnosed with ASD and their healthy siblings were recruited from the Child Development Unit at Children’s Hospital Colorado. Unrelated age-matched healthy controls were recruited from the Denver area by word of mouth. All children in the ASD cohort met criteria for an ASD based on standardized testing using the autism diagnostic observation schedule (ADOS). Individuals were excluded if they had taken an antibiotic in the last 6 months or had comorbidities, including Fragile X, Down syndrome, 22q11 deletion syndrome, neurofibromatosis, tuberous sclerosis, or metabolic syndromes. Informed consent of parents and assent of children were obtained under guidelines of CoMIRB protocol 11-0893. Between 1 and 4 stool samples were collected from each study participant spaced an average of 6 months (SD = 3 months) apart (span of 3 to 13 months; Table 1). Stool samples were collected at home by study participants in provided sterile containers and shipped overnight with 20°C freezer packs to the University of Colorado. Samples were then stored at −80°C before DNA extraction and sequencing. DNA extraction was performed at the site of sample collection (CO or AZ) with the same protocol (MoBio PowerSoil DNA isolation kit [Qiagen, Venlo, Limburg, NL]). Study participants filled out questionnaires at the time of each stool sample collection (additional details on these questionnaires follow). These included (i) the ABC, which is a validated tool that characterizes behavioral symptoms of children with atypical behavior (9) and which was used to evaluate behavior for 4 weeks prior to sample collection, (ii) the Block Kids 2004 FFQ (56), which assessed diet over the week prior to sample collection, and (iii) a GI symptoms questionnaire asking study participants to report changes in diet and frequency of constipation, diarrhea, bloating, and abdominal pain over the past 3 months or since the previous sample.

**Arizona.** Samples from individuals previously recruited for 2013 and 2018 studies by Kang et al. (23, 24), including 38 neurotypical children and 36 children with ASD, were used for the cross-sectional analyses. The subjects spanned the ages of 3 to 17 and were not using antifungals or antibiotics for at least 1 month prior to sample collection. For detailed information about subject recruitment, see Materials and Methods from previous studies.

### Gastrointestinal symptoms questionnaire.

The AZ questionnaire for participation in Autism Gut Microbiome Study by the Bhare Foundation asked study participants to report the frequency of constipation, diarrhea, average stool consistency, stool smell, flatulence, and abdominal pain within 1 week of producing their sample. Frequency of constipation, average stool consistency, stool smell, flatulence, and abdominal pain were evaluated on a weekly basis, and diarrhea was evaluated on a daily basis. The frequencies were then scored on a scale of 1 to 2.

The CO GI symptoms questionnaire asked study participants to report the frequency of constipation, diarrhea, bloating, and abdominal pain within the 3 months prior to producing their sample or since the previous sample. Study participants were given the following frequency choices for each GI symptom: less than 1 time per month, 1 to 2 times per month, 3 to 4 times per month, 1 to 2 times per week, and greater than 3 times per week for each symptom.

Responses to questionnaires were standardized for use in the cross-sectional analysis by assigning a sub score to the frequency responses from the CO GI symptom questionnaire using the same scale as that of the AZ GI questionnaire for constipation, diarrhea, and abdominal pain. Frequency reported less than once a week was assigned a score of zero. Frequency reported once to twice a week was assigned a score of 1. Frequency reported more than 3 times a week was assigned a score of 2. An overall GI symptom score was then assigned to each study participant by summation of these sub scores. CO additionally collected information on diet and behavior that is described in “Longitudinal sequence analysis.”

### Dietary questionnaire.

To understand the composition of each individual’s diet for the week prior to fecal sample collection, the FFQ was completed by the parent(s) of the study participants. The questionnaire can be completed in 25 min and contains questions about consumption of 77 food items, and pictures are provided to improve portion size reporting. NHANES 1999 to 2002 (https://www.cdc.gov/nchs/nhanes/) dietary recall data were used to develop the food list in the questionnaire. After completion of the questionnaire, dietary analysis output was determined by Nutrition Quest (Berkeley, CA, USA) and returned to us for downstream analyses.

### Aberrant behavior checklist.

Each ASD study participant’s parent(s) filled out an ABC, assessing the child’s behavior for the 4 weeks prior to providing a fecal sample. The ABC investigates 58 behavioral items and is a 5-factor scale where a larger value equals more severe atypical behavior for the preceding 4 weeks. The five factors assessed were hyperactivity, inappropriate speech, irritability, lethargy/social withdrawal, and stereotypy.

### DNA sequencing protocol.

Stool samples were stored at −80°C until DNA extraction. Genomic DNA was extracted using the MoBio PowerSoil DNA isolation kit (Qiagen, Venlo, Limburg, NL). 16S rRNA genes were amplified via PCR in triplicate using barcoded PCR primers with 5PRIME HotMasterMix (Quantabio, Beverly, MA, USA) for each sample according to the Earth Microbiome Project (EMP) protocol (57). DNA extracted in CO was sent to AZ to be sequenced together with the AZ samples. Additionally, 9 samples from CO were sent to AZ for extraction as a control by employing the same isolation kit and protocols as those used in CO. Next-generation sequencing of the V4 region of the 16S rRNA gene region was performed on extracted DNA on the Illumina MiSeq platform (two MiSeq runs).

### Cross-sectional sequence analysis.

Quantitative Insights Into Microbial Ecology (QIIME) 2 (v2019.1) (58) was used to do the cross-sectional analysis of CO and AZ locations. Keemei was used in Google documents for mapping file validation (59). Once validated, both sequencing runs were demultiplexed independently and sorted according to the sample’s corresponding barcodes, and quality control was done using DADA2 (27) to denoise the reads, remove chimeric sequences, dereplicate sequences, and define ASVs. Samples were trimmed using the following lengths. Forward reads: truncation length, 149; left trim, 10. Only the forward reads that had a Q score of 30 or above were used. ASVs were assigned taxonomically using the RDP Classifier (60) and the Greengenes database version 13.8 and binned into genera using QIIME 2. All samples, split between two runs, yielded 16,448,151 sequences, and after quality control the total of remaining sequences was 15,135,213, with an average of 93,693 reads per sample for run 1 and 59,606 for run 2. A phylogenetic tree for diversity analysis was built using MAFFT (61) and FastTree (62) (QIIME 2; qiime phylogeny align-to-tree-mafft-fasttree). Samples were rarefied at 6,529 sequences for beta and alpha diversity analyses.

Random Forest was run using QIIME 2 (v2019.7; qiime sample-classifier classify-samples) using either ASVs or genera as predictors. For the ASV sample classifier, 1,411 features were used, and for the genus sample classifier, 226 genera were used. The input samples were randomly split into a training set containing 80% of the samples and a test set used to estimate the model accuracy containing 20% of the samples. Five-fold cross-validations were performed and used to calculate an average AUC for the ASV and genera predictors.

### Choosing representative samples among longitudinal data for Colorado.

Individuals from CO, and not AZ, provided multiple samples, so a representative sample was selected for the cross-sectional analysis. Weighted and unweighted UniFrac distance matrices were used to create PCoA plots of CO samples (QIIME 1, core_diversity_analyses.py). Emperor (63) was used to generate vectors between all time points of stool samples for each study participant (QIIME 1, make_emperor.py with add_vectors flag). Both PCoA plots were analyzed to choose a representative sample. Each study participant’s sample was viewed individually in Emperor, and the most central sample among the time points was chosen. For individuals that returned only 2 samples, the first returned sample was chosen.

### Longitudinal sequence analysis.

Analysis of the longitudinal Colorado next-generation sequences was performed independently from the cross-sectional portion of this analysis using QIIME 2 (v2017.9 through v2017.11) (58). CO longitudinal samples, split between two runs, yielded 4,818,476 (run 1) and 1,805,721 (run 2) sequences, and after quality control the remaining sequence totals were 3,714,950 (run 1) and 1,017,263 (run 2). In total, 4,732,213 sequences out of 6,624,197 sequences (71.4%) remained. Keemei was used with Google Sheets for mapping file validation (59). Both sequencing runs were demultiplexed independently and sorted according to the samples’ corresponding barcodes (QIIME 2; qiime demux emp-paired). DADA2 (27) was used to denoise the reads, remove chimeric sequences, dereplicate sequences, and trim using the following lengths for the forward reads: truncation length 148 and left trim 4. Reverse read truncation length was 147 and left trim was 1 (QIIME 2, qiime dada2 denoised-paired). Runs 1 and 2 were merged to create 1 feature table containing ASV counts and 1 representative sequences file (QIIME 2; qiime feature-table merge, qiime feature-table merge-seqs). Samples were rarefied at 17,000 sequences using the merged feature table from runs 1 and 2 (QIIME 2; qiime feature-table merge and qiime feature-table rarefy). A phylogenetic tree for diversity analysis was built using MAFFT and FastTree (QIIME 2; qiime phylogeny align-to-tree-mafft-fasttree). For volatility we looked at paired distances using qualitative and quantitative beta diversity distance matrices (QIIME 2; qiime q2-longitudinal pairwise-distances).

### Linear mixed effects model.

Distance matrices were created using qiime metadata distance-matrix for one category or qiime diversity beta (with the “euclidean” metric) for inputting a feature table with multiple categories in QIIME 2. Distance matrices for beta diversity metrics were created using weighted UniFrac and unweighted UniFrac metrics (QIIME 2; qiime diversity core-metrics-phylogenetic). Eleven Euclidean distances were created for the following: (1) ASD-associated behavioral severity for ASD individuals only, (2 to 3) GI distress in ASD and NT individuals, and (4 to 11) dietary percentages, daily totals, fiber, and percent carbohydrates for ASD and NT individuals. The overall ASD-associated behavioral severity EDM was created using the five autism behavioral severity metrics based on the ABC, which were hyperactivity, inappropriate speech, irritability, lethargy/social withdrawal, and stereotypy. The dietary percentages EDM was created based on an individual’s average daily percent consumption of fat, protein, carbohydrates, and sweets for the past week. For average daily total values, the EDM was created from dietary analysis output variables returned to us from Nutrition Quest (Table S1).

10.1128/mSystems.00848-20.6 TABLE S1 Dietary analysis output variables used for “daily totals” Euclidean distance matrix. Download Table S1, DOCX file, 0.01 MB. Copyright © 2021 Fouquier et al. 2021 Fouquier et al. https://creativecommons.org/licenses/by/4.0/ This content is distributed under the terms of the Creative Commons Attribution 4.0 International license.

For GI distress, an EDM was created using abdominal pain, bloating, constipation, or loose stool quantified for 3 months prior to producing their sample or since the previous sample.

Vectors were then created from each distance matrix by selecting within-individual comparisons and excluding those distances which compared different individuals. For all ASD-associated behavioral severity, diet, and GI EDM vector comparisons to alpha diversity, the pairwise change in Pielou’s evenness, Faith’s PD, observed OTUs, and Shannon diversity between sampling days was used. For the five individual ASD-associated behavioral severity metrics, the original behavioral severity scores were used and compared to raw Pielou’s evenness, Faith’s PD, observed OTUs, and Shannon diversity alpha diversity metrics.

For LME modeling, random intercepts were allowed for baseline subject differences and random slopes were used to allow variability in the relationship strength between response and explanatory variables for different individuals (64), as it is likely that gut microbiome relationships vary between individuals. There were no obvious departures from homoscedasticity or normality in the residual plots. We compared full and reduced models using analysis of variance (ANOVA) to obtain *P* values.

Full model: response.variable ∼ explanatory.variable + (1 + explanatory.variable|subject).

Reduced model: response.variable ∼ (1 + explanatory.variable|subject).

### Data availability.

The data sets generated and/or analyzed during the current study are available in the asd-analysis github repository (https://github.com/JTFouquier/asd-analysis). Raw sequence information can be obtained using EBI PRJEB42687.
