# Supplementary figures and images for: The Gut Microbiome in Autism: Study-Site Effects and Longitudinal Analysis of Behavior Change
Source: mSystems. 2021 Apr 6;6(2):e00848-20. doi: 10.1128/mSystems.00848-20 (PMC8546984; doi:10.1128/mSystems.00848-20)

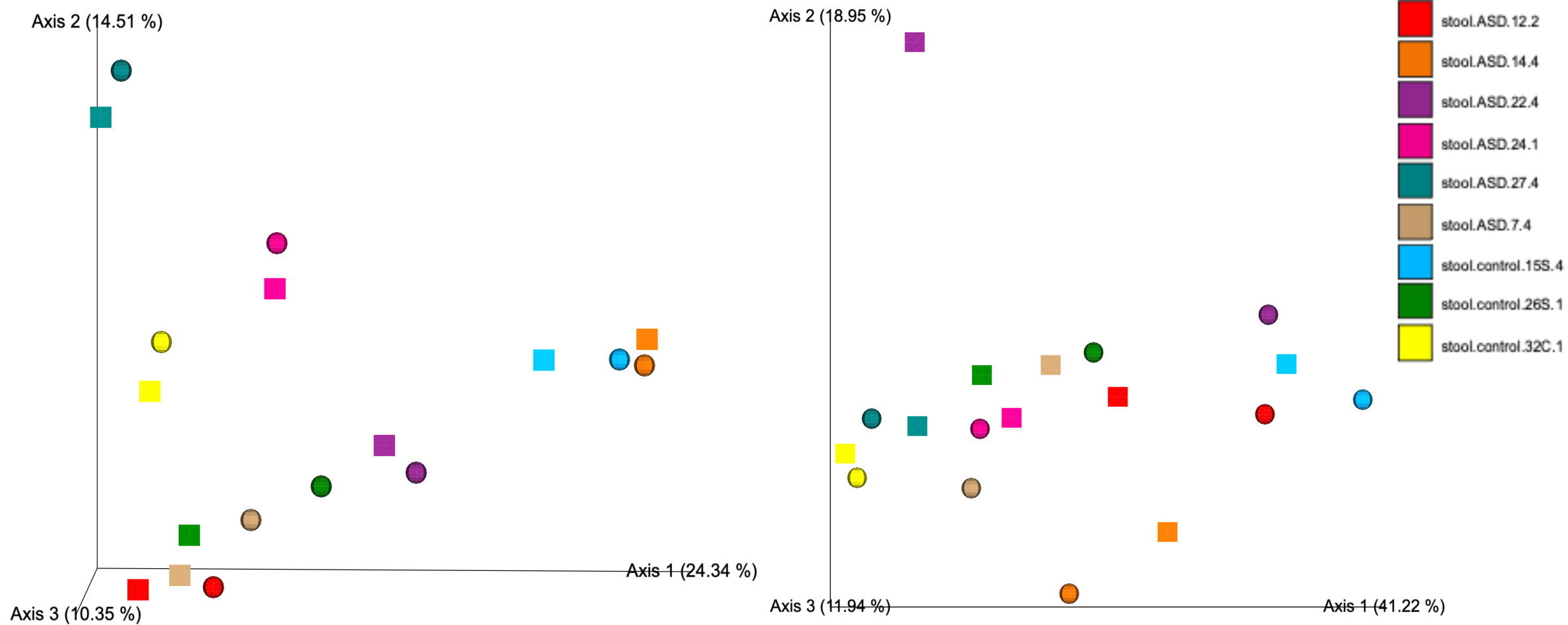

Supplement: FIG S1 [file msystems.00848-20-sf001.pdf]

PC 2 (7.987 %)

a)

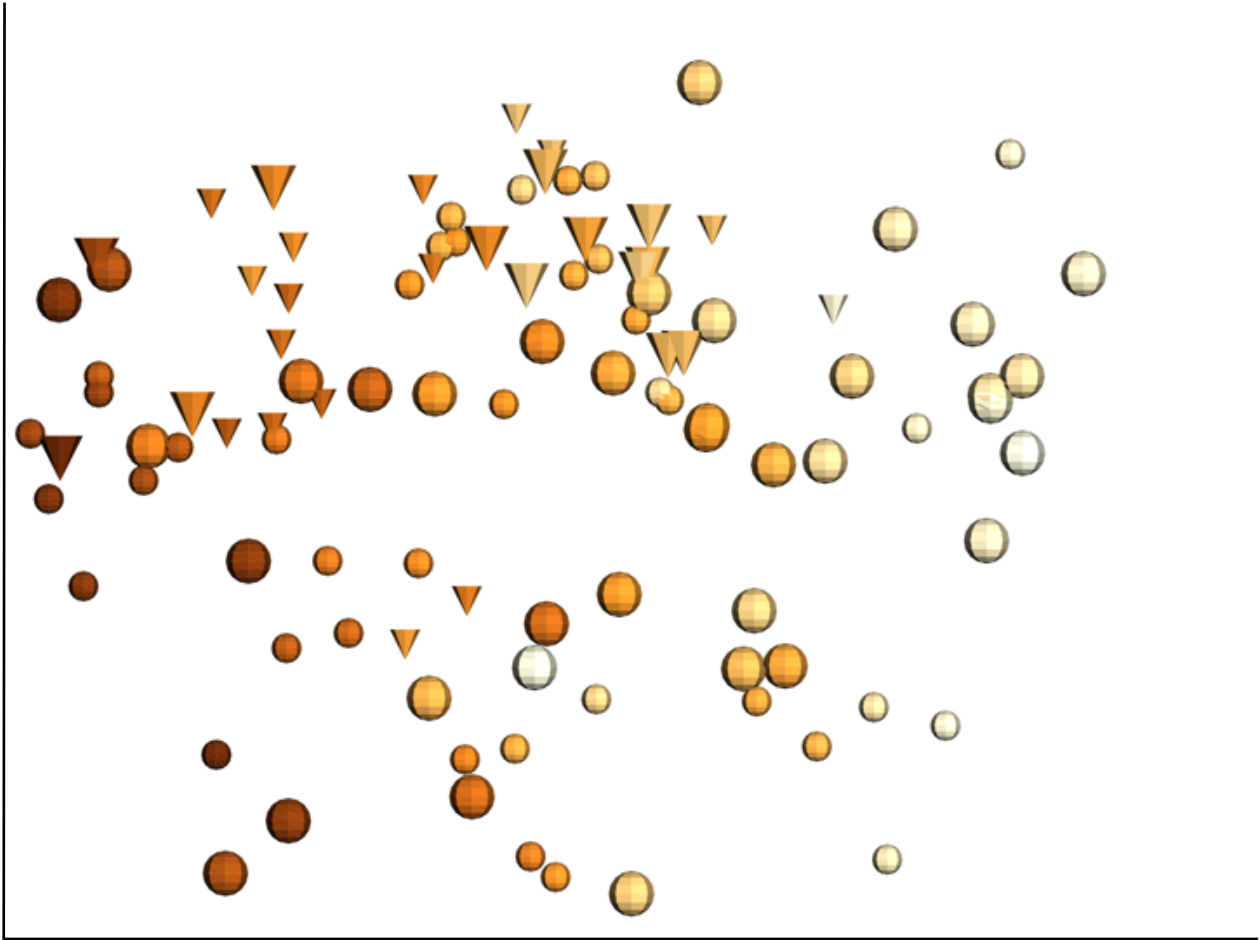

Shape

▼ CO  
● AZ

Scale

▼ ● NT  
● ▼ ASD

Color Gradient

Low Observed OTUs  
High Observed OTUs

PC 1 (14.00 %)

b)

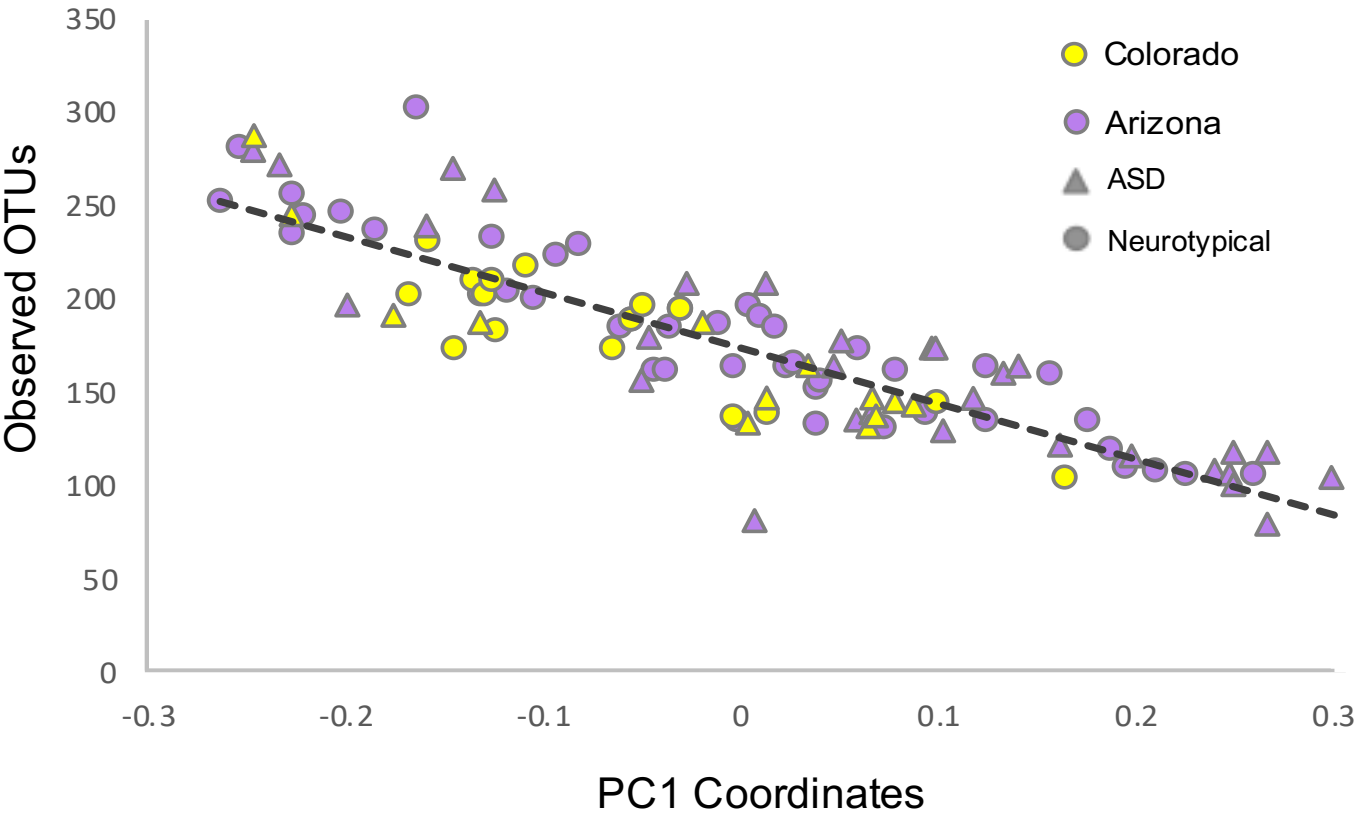

Supplement: FIG S2 [file msystems.00848-20-sf002.pdf]

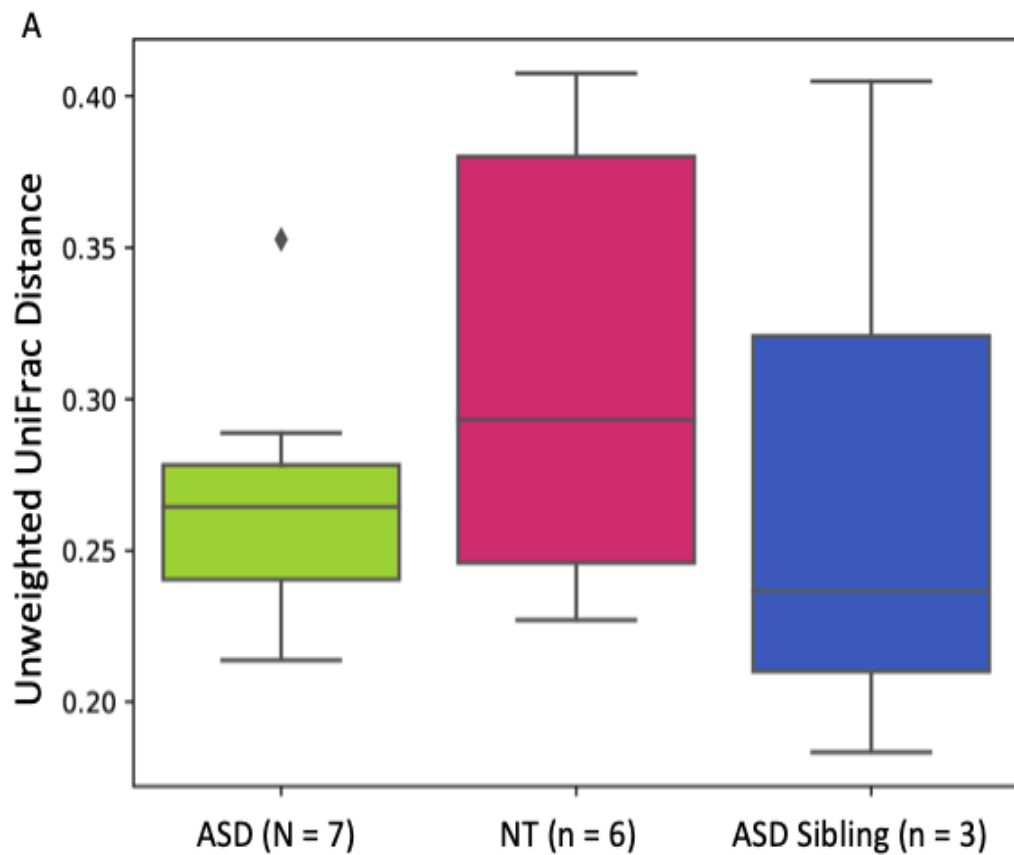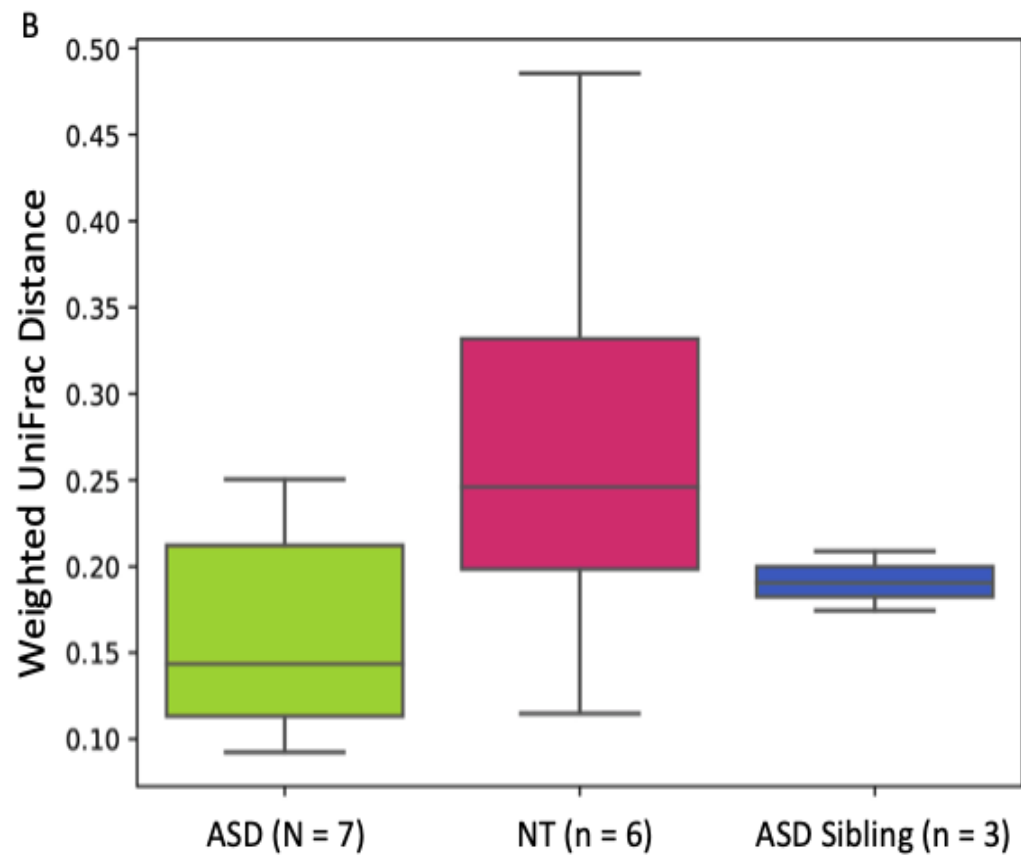

Supplement: FIG S3 [file msystems.00848-20-sf003.pdf]

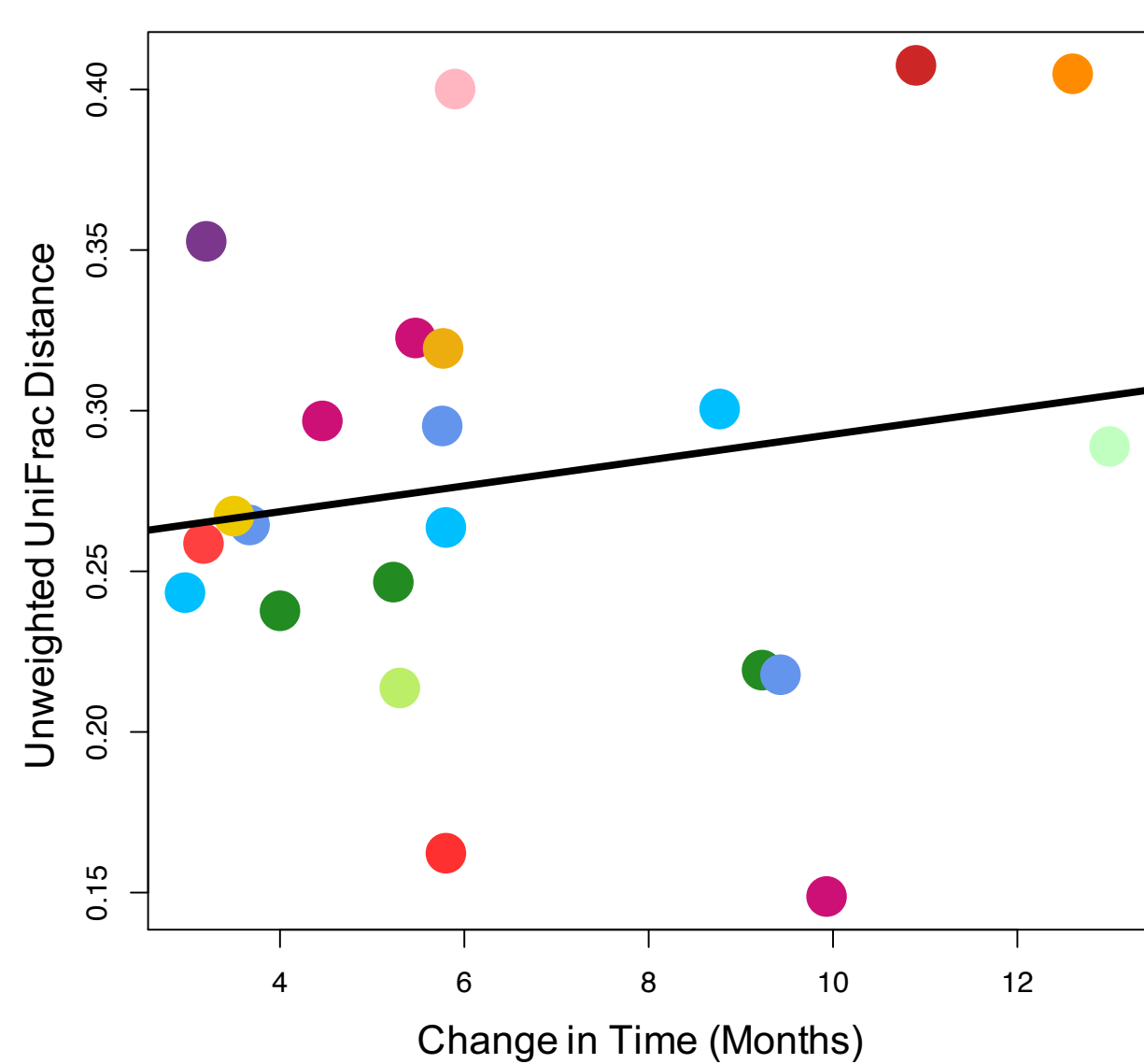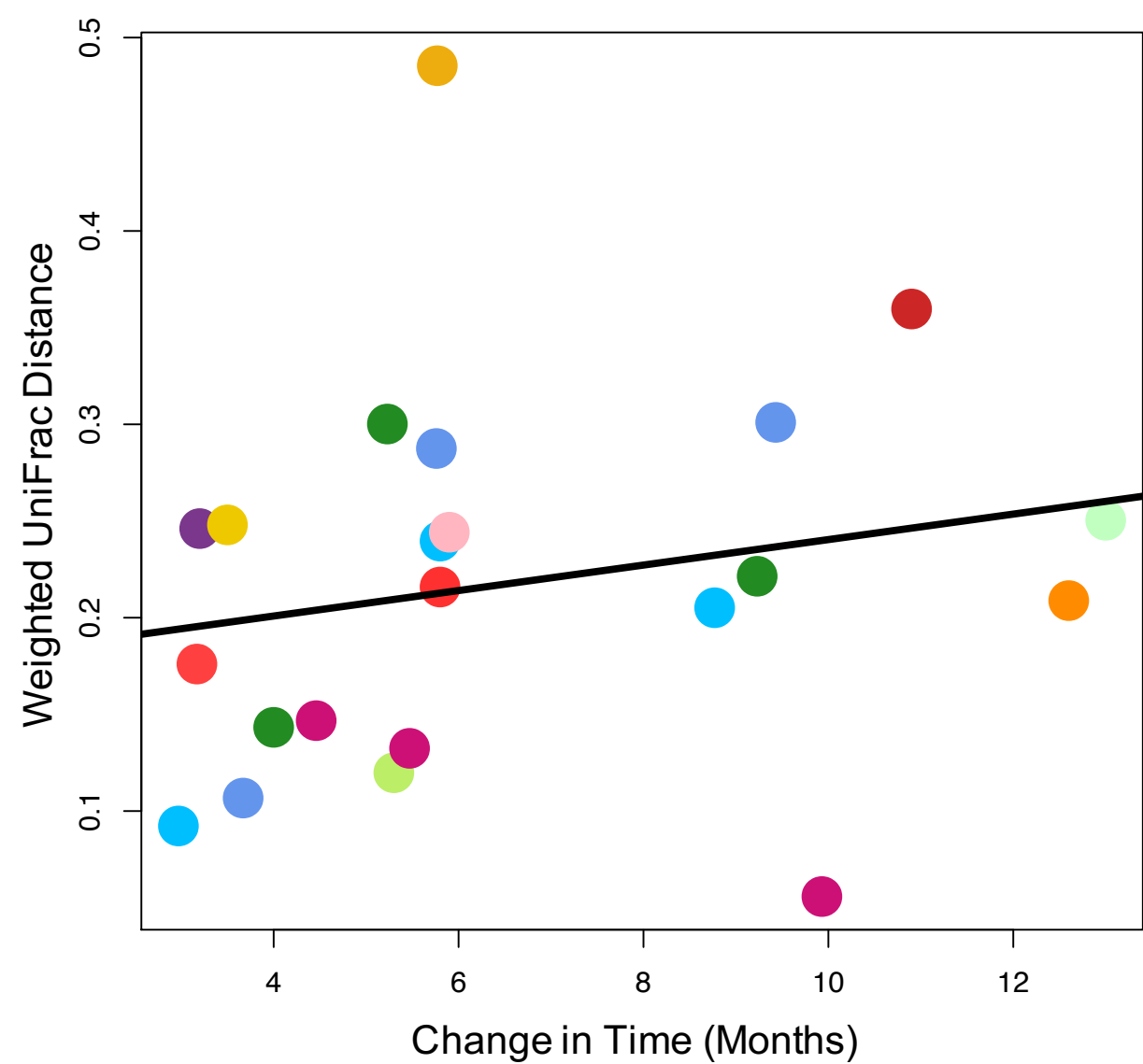

Supplement: FIG S4 [file msystems.00848-20-sf004.pdf]

# Dietary Percentages

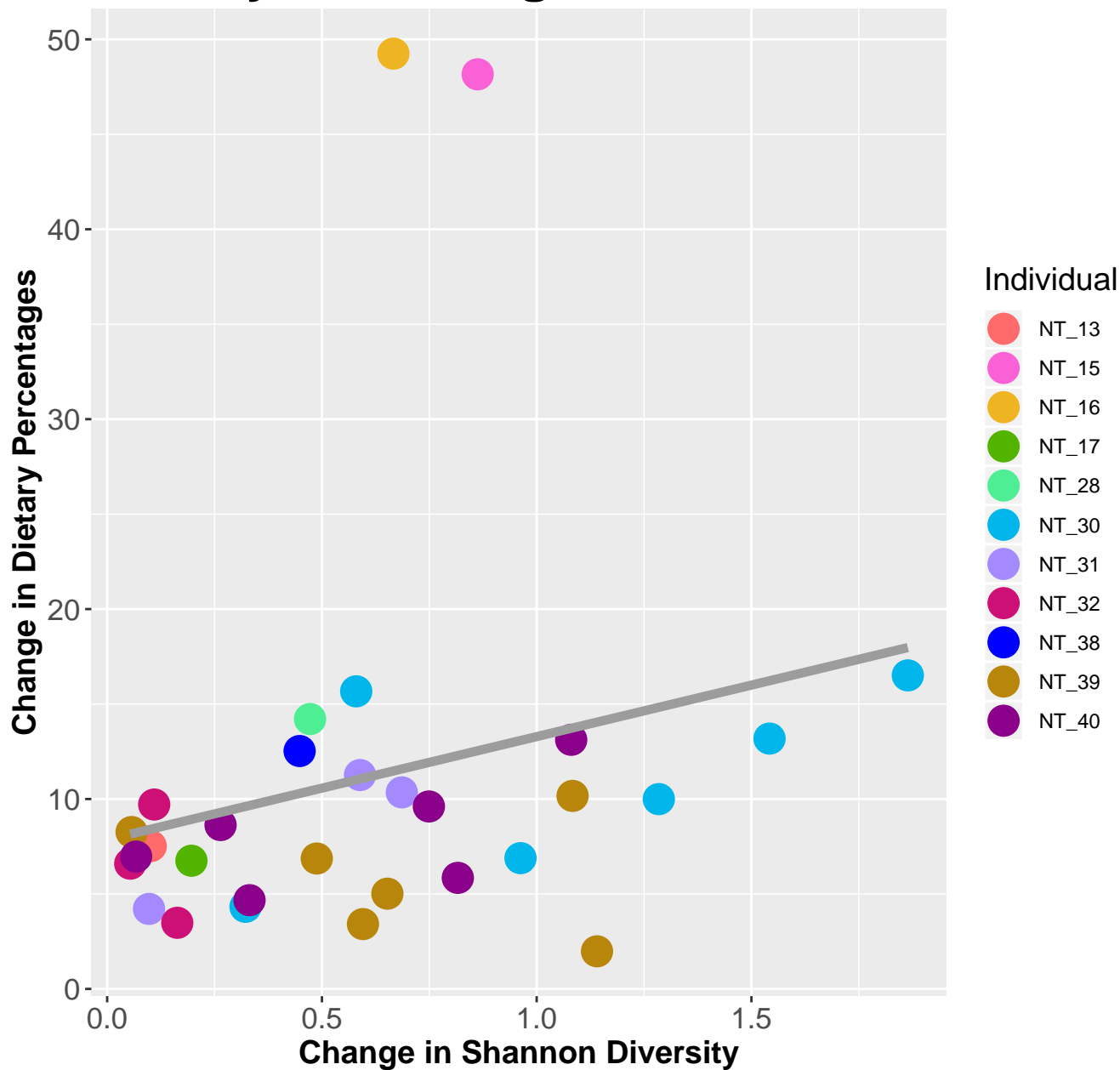

Supplement: FIG S5 [file msystems.00848-20-sf005.pdf]
