# Supplementary material for: The Gut Microbiome in Autism: Study-Site Effects and Longitudinal Analysis of Behavior Change
Source: mSystems. 2021 Apr 6;6(2):e00848-20. doi: 10.1128/mSystems.00848-20 (PMC8546984; doi:10.1128/mSystems.00848-20)
Supplement: TABLE S3 [file msystems.00848-20-st003.docx]

**Table S3.** Important features from ASV and Genus Random Forest classifiers created using combined Arizona and Colorado samples. Most important features are at the top and listed in descending order using a cutoff of 0.01.

| **ASV Classifier** | Random Forest Importance |
| --- | --- |
| p__Firmicutes.c__Clostridia.o__Clostridiales.f__.g__.s__ | 0.049 |
| p__Firmicutes.c__Clostridia.o__Clostridiales.f__Lachnospiraceae.g__.s__ | 0.036 |
| p__Firmicutes.c__Clostridia.o__Clostridiales.f__Lachnospiraceae.g__Blautia.s__ | 0.022 |
| p__Firmicutes.c__Clostridia.o__Clostridiales.f__.g__.s__ | 0.021 |
| p__Firmicutes.c__Clostridia.o__Clostridiales.f__Clostridiaceae.g__Sarcina.s__ | 0.021 |
| p__Bacteroidetes.c__Bacteroidia.o__Bacteroidales.f__Rikenellaceae.g__Alistipes.s__putredinis | 0.019 |
| p__Bacteroidetes.c__Bacteroidia.o__Bacteroidales.f__Bacteroidaceae.g__Bacteroides.s__fragilis | 0.016 |
| p__Firmicutes.c__Clostridia.o__Clostridiales.f__Lachnospiraceae | 0.014 |
| p__Firmicutes.c__Clostridia.o__Clostridiales.f__Lachnospiraceae.g__Blautia | 0.014 |
| p__Bacteroidetes.c__Bacteroidia.o__Bacteroidales.f__Bacteroidaceae.g__Bacteroides.s__ovatus | 0.013 |
| p__Bacteroidetes.c__Bacteroidia.o__Bacteroidales.f__Prevotellaceae.g__Prevotella.s__copri | 0.012 |
| p__Firmicutes.c__Clostridia.o__Clostridiales.f__Lachnospiraceae.g__Coprococcus.s__ | 0.011 |
| p__Firmicutes.c__Clostridia.o__Clostridiales.f__Lachnospiraceae.g__Roseburia.s__ | 0.010 |
|  |  |
| **Genus Classifier** |  |
| p__Firmicutes.c__Erysipelotrichi.o__Erysipelotrichales.f__Erysipelotrichaceae.g__ | 0.029 |
| p__Firmicutes.c__Clostridia.o__Clostridiales.f__.g__ | 0.027 |
| p__Firmicutes.c__Clostridia.o__Clostridiales.f__Lachnospiraceae.g__Blautia | 0.025 |
| p__Firmicutes.c__Erysipelotrichi.o__Erysipelotrichales.f__Erysipelotrichaceae.g__Clostridium | 0.023 |
| p__Firmicutes.c__Clostridia.o__Clostridiales.f__Clostridiaceae.g__Clostridium | 0.021 |
| p__Actinobacteria.c__Coriobacteriia.o__Coriobacteriales.f__Coriobacteriaceae.g__Eggerthella | 0.020 |
| p__Verrucomicrobia.c__Verrucomicrobiae.o__Verrucomicrobiales.f__Verrucomicrobiaceae.g__Akkermansia | 0.020 |
| p__Proteobacteria.c__Betaproteobacteria.o__Burkholderiales.f__Alcaligenaceae.g__Sutterella | 0.020 |
| p__Firmicutes.c__Clostridia.o__Clostridiales.f__Ruminococcaceae.g__ | 0.019 |
| p__Bacteroidetes.c__Bacteroidia.o__Bacteroidales.f__Bacteroidaceae.g__Bacteroides | 0.019 |
| p__Firmicutes.c__Clostridia.o__Clostridiales.f__Christensenellaceae.g__ | 0.019 |
| p__Firmicutes.c__Clostridia.o__Clostridiales.f__Lachnospiraceae.g__Coprococcus | 0.018 |
| p__Firmicutes.c__Clostridia.o__Clostridiales.f__Clostridiaceae.g__Sarcina | 0.018 |
| p__Firmicutes.c__Clostridia.o__Clostridiales.f__Lachnospiraceae.g__Dorea | 0.018 |
| p__Firmicutes.c__Clostridia.o__Clostridiales.f__Lachnospiraceae.g__ | 0.017 |
| p__Firmicutes.c__Clostridia.o__Clostridiales.f__.Mogibacteriaceae..g__ | 0.017 |
| p__Firmicutes.c__Clostridia.o__Clostridiales.f__Ruminococcaceae.g__Gemmiger | 0.017 |
| p__Firmicutes.c__Bacilli.o__Lactobacillales.f__Streptococcaceae.g__Streptococcus | 0.016 |
| p__Bacteroidetes.c__Bacteroidia.o__Bacteroidales.f__Rikenellaceae.g__Alistipes | 0.016 |
| p__Actinobacteria.c__Actinobacteria.o__Bifidobacteriales.f__Bifidobacteriaceae.g__Bifidobacterium | 0.016 |
| p__Firmicutes.c__Clostridia.o__Clostridiales.f__Clostridiaceae.g__ | 0.015 |
| p__Firmicutes.c__Clostridia.o__Clostridiales.f__Peptostreptococcaceae.__ | 0.015 |
| p__Firmicutes.c__Clostridia.o__Clostridiales.f__Lachnospiraceae.__ | 0.015 |
| p__Bacteroidetes.c__Bacteroidia.o__Bacteroidales.f__Porphyromonadaceae.g__Parabacteroides | 0.014 |
| p__Bacteroidetes.c__Bacteroidia.o__Bacteroidales.f__Prevotellaceae.g__Prevotella | 0.014 |
| p__Firmicutes.c__Bacilli.o__Turicibacterales.f__Turicibacteraceae.g__Turicibacter | 0.014 |
| p__Firmicutes.c__Clostridia.o__Clostridiales.f__Ruminococcaceae.__ | 0.014 |
| p__Proteobacteria.c__Gammaproteobacteria.o__Enterobacteriales.f__Enterobacteriaceae.__ | 0.014 |
| p__Firmicutes.c__Clostridia.o__Clostridiales.__.__ | 0.014 |
| p__Firmicutes.c__Clostridia.o__Clostridiales.f__Lachnospiraceae.g__Clostridium | 0.013 |
| p__Actinobacteria.c__Coriobacteriia.o__Coriobacteriales.f__Coriobacteriaceae.g__ | 0.013 |
| p__Firmicutes.c__Erysipelotrichi.o__Erysipelotrichales.f__Erysipelotrichaceae.g__.Eubacterium. | 0.013 |
| p__Firmicutes.c__Clostridia.o__Clostridiales.f__Ruminococcaceae.g__Faecalibacterium | 0.013 |
| p__Firmicutes.c__Clostridia.o__Clostridiales.f__Ruminococcaceae.g__Ruminococcus | 0.013 |
| p__Firmicutes.c__Clostridia.o__Clostridiales.f__Ruminococcaceae.g__Oscillospira | 0.013 |
| p__Firmicutes.c__Clostridia.o__Clostridiales.f__Lachnospiraceae.g__Roseburia | 0.012 |
| p__Firmicutes.c__Clostridia.o__Clostridiales.f__Lachnospiraceae.g__Lachnospira | 0.012 |
| p__Firmicutes.c__Clostridia.o__Clostridiales.f__Lachnospiraceae.g__.Ruminococcus. | 0.012 |
| p__Actinobacteria.c__Coriobacteriia.o__Coriobacteriales.f__Coriobacteriaceae.g__Collinsella | 0.012 |
| p__Firmicutes.c__Erysipelotrichi.o__Erysipelotrichales.f__Erysipelotrichaceae.g__Holdemania | 0.012 |
| p__Firmicutes.c__Clostridia.o__Clostridiales.f__Lachnospiraceae.g__Anaerostipes | 0.012 |
