# Supplementary material for: The Gut Microbiome in Autism: Study-Site Effects and Longitudinal Analysis of Behavior Change
Source: mSystems. 2021 Apr 6;6(2):e00848-20. doi: 10.1128/mSystems.00848-20 (PMC8546984; doi:10.1128/mSystems.00848-20)
Supplement: TABLE S2 [file msystems.00848-20-st002.docx]

**Table S2.** Summary of linear mixed effect models (LMEs) relating autism behavioral severity metrics, GI distress, and diet to unweighted UniFrac and weighted UniFrac beta diversity microbiome metrics and Shannon diversity, observed OTUs, Evenness and Faith's PD alpha diversity microbiome metrics.

|  | Beta Diversity | | | | | | | | Alpha Diversity | | | | | | | | | | | | | | | |
| --- | --- | --- | --- | --- | --- | --- | --- | --- | --- | --- | --- | --- | --- | --- | --- | --- | --- | --- | --- | --- | --- | --- | --- | --- |
|  | Unweighted UniFrac | | | | Weighted UniFrac | | | | Shannon Diversity | | | | Observed OTUs | | | | Evenness | | | | Faith's PD | | | |
|  | Est. | χ2 | p | q | Est. | χ2 | p | q | Est. | χ2 | p | q | Est. | χ2 | p | q | Est. | χ2 | p | q | Est. | χ2 | p | q |
| ASD Behavioral Severity Metrics |  |  |  |  |  |  |  |  |  |  |  |  |  |  |  |  |  |  |  |  |  |  |  |  |
| Aberrant Behavior Checklist EDM | ⎯ | ⎯ | ns | ⎯ | ⎯ | ⎯ | ns | ⎯ | ⎯ | ⎯ | ns | ⎯ | ⎯ | ⎯ | ns | ⎯ | ⎯ | ⎯ | ns | ⎯ | ⎯ | ⎯ | ns | ⎯ |
| Hyperactivity | ⎯ | ⎯ | ns | ⎯ | ⎯ | ⎯ | ns | ⎯ | ⎯ | ⎯ | ns | ⎯ | ⎯ | ⎯ | ns | ⎯ | ⎯ | ⎯ | ns | ⎯ | ⎯ | ⎯ | ns | ⎯ |
| Inappropriate Speech | ⎯ | ⎯ | ns | ⎯ | ⎯ | ⎯ | ns | ⎯ | ⎯ | ⎯ | ns | ⎯ | -0.04 | 4.57 | 0.03 | 0.16 | ⎯ | ⎯ | ns | ⎯ | ⎯ | ⎯ | ns | ⎯ |
| Irritability | ⎯ | ⎯ | ns | ⎯ | ⎯ | ⎯ | ns | ⎯ | ⎯ | ⎯ | ns | ⎯ | ⎯ | ⎯ | ns | ⎯ | ⎯ | ⎯ | ns | ⎯ | ⎯ | ⎯ | ns | ⎯ |
| Lethargy | 65.72 | 10.43 | <0.001 | 0.01 | ⎯ | ⎯ | ns | ⎯ | ⎯ | ⎯ | ns | ⎯ | ⎯ | ⎯ | ns | ⎯ | ⎯ | ⎯ | ns | ⎯ | ⎯ | ⎯ | ns | ⎯ |
| Stereotypy | ⎯ | ⎯ | ns | ⎯ | ⎯ | ⎯ | ns | ⎯ | ⎯ | ⎯ | ns | ⎯ | ⎯ | ⎯ | ns | ⎯ | ⎯ | ⎯ | ns | ⎯ | ⎯ | ⎯ | ns | ⎯ |
| GI Distress |  |  |  |  |  |  |  |  |  |  |  |  |  |  |  |  |  |  |  |  |  |  |  |  |
| ASD GI distress EDM | ⎯ | ⎯ | ns | ⎯ | ⎯ | ⎯ | ns | ⎯ | ⎯ | ⎯ | ns | ⎯ | ⎯ | ⎯ | ns | ⎯ | ⎯ | ⎯ | ns | ⎯ | ⎯ | ⎯ | ns | ⎯ |
| Control GI distress EDM | ⎯ | ⎯ | ns | ⎯ | ⎯ | ⎯ | ns | ⎯ | ⎯ | ⎯ | ns | ⎯ | ⎯ | ⎯ | ns | ⎯ | ⎯ | ⎯ | ns | ⎯ | ⎯ | ⎯ | ns | ⎯ |
| Diet |  |  |  |  |  |  |  |  |  |  |  |  |  |  |  |  |  |  |  |  |  |  |  |  |
| ASD DTs EDM | ⎯ | ⎯ | ns | ⎯ | ⎯ | ⎯ | ns | ⎯ | ⎯ | ⎯ | ns | ⎯ | ⎯ | ⎯ | ns | ⎯ | ⎯ | ⎯ | ns | ⎯ | ⎯ | ⎯ | ns | ⎯ |
| ASD Percents EDM | ⎯ | ⎯ | ns | ⎯ | ⎯ | ⎯ | ns | ⎯ | ⎯ | ⎯ | ns | ⎯ | ⎯ | ⎯ | ns | ⎯ | ⎯ | ⎯ | ns | ⎯ | ⎯ | ⎯ | ns | ⎯ |
| ASD Fiber EDM | ⎯ | ⎯ | ns | ⎯ | ⎯ | ⎯ | ns | ⎯ | ⎯ | ⎯ | ns | ⎯ | ⎯ | ⎯ | ns | ⎯ | ⎯ | ⎯ | ns | ⎯ | ⎯ | ⎯ | ns | ⎯ |
| ASD Carbohydrates EDM | ⎯ | ⎯ | ns | ⎯ | ⎯ | ⎯ | ns | ⎯ | ⎯ | ⎯ | ns | ⎯ | ⎯ | ⎯ | ns | ⎯ | ⎯ | ⎯ | ns | ⎯ | ⎯ | ⎯ | ns | ⎯ |
| NT Control DTs EDM | ⎯ | ⎯ | ns | ⎯ | ⎯ | ⎯ | ns | ⎯ | ⎯ | ⎯ | ns | ⎯ | ⎯ | ⎯ | ns | ⎯ | ⎯ | ⎯ | ns | ⎯ | ⎯ | ⎯ | ns | ⎯ |
| NT Control Percents EDM | ⎯ | ⎯ | ns | ⎯ | ⎯ | ⎯ | ns | ⎯ | 15.76 | 8.31 | <0.001 | 0.02 | ⎯ | ⎯ | ns | ⎯ | ⎯ | ⎯ | ns | ⎯ | ⎯ | ⎯ | ns | ⎯ |
| NT Control Fiber EDM | ⎯ | ⎯ | ns | ⎯ | ⎯ | ⎯ | ns | ⎯ | ⎯ | ⎯ | ns | ⎯ | ⎯ | ⎯ | ns | ⎯ | ⎯ | ⎯ | ns | ⎯ | ⎯ | ⎯ | ns | ⎯ |
| NT Control Carbohydrates EDM | ⎯ | ⎯ | ns | ⎯ | ⎯ | ⎯ | ns | ⎯ | ⎯ | ⎯ | ns | ⎯ | ⎯ | ⎯ | ns | ⎯ | ⎯ | ⎯ | ns | ⎯ | ⎯ | ⎯ | ns | ⎯ |

All Euclidean distance matrices (EDMs) were either compared to another distance matrix or absolute change in alpha diversity. If metric was not a distance matrix, original alpha diversity values were used. When significant p-values <0.05 were obtained, FDR-adjusted q-values, estimate, and chi-squared value were reported when comparing the full and reduced model using ANOVA.
