# Supplementary material for: The Gut Microbiome in Autism: Study-Site Effects and Longitudinal Analysis of Behavior Change
Source: mSystems. 2021 Apr 6;6(2):e00848-20. doi: 10.1128/mSystems.00848-20 (PMC8546984; doi:10.1128/mSystems.00848-20)
Supplement: TABLE S1 [file msystems.00848-20-st001.docx]

| **TABLE S1:** Dietary analysis output variables used for “daily totals” Euclidean distance matrix | | |
| --- | --- | --- |
| Food energy, kcals | Dietary fiber, gm | Selenium, mcg |
| Protein, gm | Food folate, mcg | Dietary Folic acid from fortification, mcg |
| Fat, gm | Zinc, total, mg | Dietary folate (natural, from food), mcg |
| Carbohydrate, gm | Zinc, animal sources only, mg | Total folate/folic acid, mcgs Dietary folate equivalents (DFE) |
| Calcium, mg | Vitamin B6, mg | Dietary Vitamin K, mcg |
| Phosphorus, mg | Magnesium, mg | Dietary theobromine, mgs |
| Iron, mg | Alpha-carotene, mcg | Sugars, total, gm |
| Sodium, mg | Beta-carotene, mcg | Fructose, gm |
| Potassium, mg | Cryptoxanthin, beta, mcg | Lactose, gm |
| Thiamin (Vitamin B1), mg | Lutein-Zeaxanthin, mcg | Maltose, gm |
| Riboflavin (Vitamin B2), mg | Lycopene, mcg | Galactose, gm |
| Niacin, mg | Retinol, mcg | Sucrose, gm |
| Vitamin C, mg | Pro-vitamin A carotenoids, mcg | Glucose, gm |
| Saturated fat, gm | Vitamin A, RAE, mcg | Omega-3 fatty acids, gm |
| Monounsaturated fatty acids, gm | Vitamin E as alpha-tocopherol, mg | Omega-6 fatty acids, gm |
| Polyunsaturated fatty acids, gm | Vitamin B-12, mcg | Vitamin D, IU |
| Cholesterol, mg | Copper, mg | Trans fats, total, gm |
